# Cognitive dysfunction in type 1 diabetes: role of TREM2 in microglial activation and Aβ pathology

**DOI:** 10.1186/s12974-025-03611-3

**Published:** 2026-01-02

**Authors:** Yue Wang, Ruyue Wang, Yimeng Liu, Zhaohui Wang, Hongyan Ding, Xinyi Wei, Aikeda Aihemaitijiang, Minghan Sun, Li Zhao

**Affiliations:** 1 Department of Neurobiology, School of Basic Medical Sciences, Capital Medical University, Beijing, 100069 PR China; 2 Beijing Key Laboratory of Mental Disorders, National Clinical Research Center for Mental Disorders & National Center for Mental Disorders, Beijing Anding Hospital, Capital Medical University, Beijing, 100088 China; 3 (Present address) Cangzhou Central Hospital, Cangzhou, Hebei 061001 China

**Keywords:** TREM2, Microglia, Type 1 diabetes, Amyloid-beta

## Abstract

**Background:**

Cognitive dysfunction associated with type 1 diabetes (T1D) is closely linked to the accumulation of amyloid-beta (Aβ) oligomers. However, the role of microglia and their underlying molecular mechanisms in this process remain unclear. Triggering receptor expressed on myeloid cells 2 (TREM2), a microglial receptor critical for clearing neurotoxic Aβ and maintaining metabolic homeostasis, is dysfunctional in Alzheimer’s disease. Here, we investigated TREM2-mediated microglial dysfunction in diabetic neurodegeneration.

**Purpose:**

To investigate the role of TREM2-mediated microglial dysfunction in Aβ clearance and cognitive impairment in T1D.

**Basic procedures:**

A total of 204 male C57BL/6J mice, aged 6–8 weeks, were used in this study. We performed single-nucleus RNA sequencing (snRNA-seq) on 59,356 cells from the prefrontal cortex and hippocampus. Aβ pathology was evaluated by western blot, immunofluorescence and ELISA. TREM2 knockout mice and the murine microglial cell line BV2 were used to study the role of TREM2 in cognitive function and Aβ clearance.

**Main findings:**

T1D mice exhibited progressive memory deficits and prefrontal Aβ oligomer accumulation (36–50 kDa), with region-specific microglial activation. SnRNA-seq identified ten microglial subpopulations, with Trem2-enriched clusters (M1/M2/M3/M5) showing impaired phagocytosis and metabolic dysregulation. TREM2 knockout exacerbated cognitive deficits and Aβ accumulation in T1D mice. Mechanistically, TREM2 regulated microglial migration, phagocytosis of Aβ oligomers, and mitochondrial integrity under high-glucose conditions, potentially via the mTOR signaling pathway.

**Principle conclusions:**

These findings establish TREM2 as a critical regulator of microglial Aβ clearance in T1D, operating mitochondrial and phagocytic programs via mTOR and highlighting its therapeutic potential for diabetic neurodegeneration.

**Supplementary Information:**

The online version contains supplementary material available at 10.1186/s12974-025-03611-3.

## Introduction

Type 1 diabetes (T1D), accounting for approximately 5% of all diabetes cases, primarily manifests in youth as a consequence of autoimmune-mediated insulin deficiency. Numerous previous studies have demonstrated that dysglycemia is closely associated with cognitive impairment. Chronic hyperglycemia can promote neuronal damage and vascular endothelial dysfunction in the brain, leading to alterations in grey matter (particularly in key regions such as the posterior cingulate cortex and hippocampus) and cognitive decline, especially in the domains of executive function, memory, and information processing speed [1–7]. Hyperglycemia drives cerebral pathological processes through various mechanisms, including induction of endoplasmic reticulum stress, neuroinflammation, immune dysregulation, and cell death. Chronic hyperglycemia can compromise blood-brain barrier integrity, thereby permitting inflammatory factors to enter the brain and indirectly provoking microglial activation [8]. Moreover, high glucose may also act directly on microglia. For example, it can activate the AGEs-RAGE-NF-κB pathway, leading to inflammation and oxidative stress, or it can reprogram glucose metabolism, driving a shift toward a pro-inflammatory phenotype [9].

Microglia, the primary immune cells in the brain, exhibit a “double-edged sword” nature, with different phenotypes exerting either detrimental or protective effects in various pathological processes [10]. For example, disease-associated microglia (DAM) are considered beneficial as they are involved in the clearance of Aβ in AD models [11]. In contrast, some reactive microglia can be detrimental when they become overactivated and release excessive pro-inflammatory cytokines and reactive oxygen species (ROS), exacerbating neuroinflammation and neuronal damage in conditions like Parkinson’s disease and amyotrophic lateral sclerosis [12, 13]. Recent single-nucleus RNA sequencing (snRNA-seq) approaches have significantly advanced the identification of novel microglial subtypes and state transitions under disease conditions. Our snRNA-seq analysis of diabetic neurodegeneration models identified metabolically compromised microglial clusters exhibiting coordinated upregulation of triggering receptor expressed on myeloid cells 2 (TREM2).

TREM2, a transmembrane immunoreceptor predominantly expressed by microglia, serves as a molecular hub coordinating phagocytic capacity, lipid metabolism, and inflammatory tone [14, 15]. TREM2 forms a signaling complex with DNAX-activation protein 12 (DAP12) through its cytoplasmic domain, initiating Syk tyrosine kinase recruitment and subsequent activation of downstream effectors including PI3K/Akt/mTOR and extracellular signal-regulated kinase 1/2 (Erk1/2) pathways [16]. This signaling cascade fundamentally regulates microglial homeostatic functions spanning phagocytic capacity, immunomodulatory secretion profiles, and cellular survival.

TREM2 has been identified as a receptor for Aβ, a hallmark of AD pathogenesis [17, 18]. Aβ is composed of 39 to 42 amino acids and exists in three main forms: monomers, oligomers and fibrils. In Alzheimer’s disease (AD), Aβ peptides transition from monomers to oligomers, then form intermediate protofibrils, and ultimately aggregate into insoluble fibrillar structures that deposit. Aβ oligomers are increasingly recognized as the principal mediators of neuronal toxicity [19–22]. These oligomers induce synaptotoxicity through NMDA receptor hyperactivation, ROS generation, and glutamatergic signaling dysregulation, precipitating synaptic stripping and neurovascular dysfunction linked to cognitive deficits [23–25]. Emerging evidence indicates that microglia exhibit dualistic responses to distinct oligomeric assemblies of Aβ—efficiently clearing soluble, lower-order oligomers through phagocytic mechanisms while paradoxically amplifying neuroinflammation in response to higher-order assemblies [26]. Critically, TREM2 knockout severely compromises the clustering of microglia around Aβ plaques, resulting in more diffuse plaque morphology, and elevated levels of soluble Aβ oligomers, ultimately markedly accelerating disease progression in AD models [11, 27, 28].

We hypothesized that disrupted TREM2 signaling serves as a critical mechanism linking chronic hyperglycemia to microglial dysfunction and aberrant Aβ accumulation in T1D, thereby contributing to cognitive decline. The primary aim of this study was to elucidate the role of TREM2-mediated microglial dysfunction in T1D-associated neurodegeneration. Specifically, we aimed to: (1) characterize the transcriptomic heterogeneity of microglia in T1D mouse brains using snRNA-seq; (2) determine the spatiotemporal relationship between Aβ oligomer accumulation, microglial activation, and cognitive decline; and (3) investigate the mechanistic role of TREM2 in regulating microglial migration, phagocytosis, and mitochondrial function under high-glucose conditions, with a particular focus on the PI3K/Akt/mTOR signaling pathway.

## Materials and methods

### Animals

**Fig. 1:**
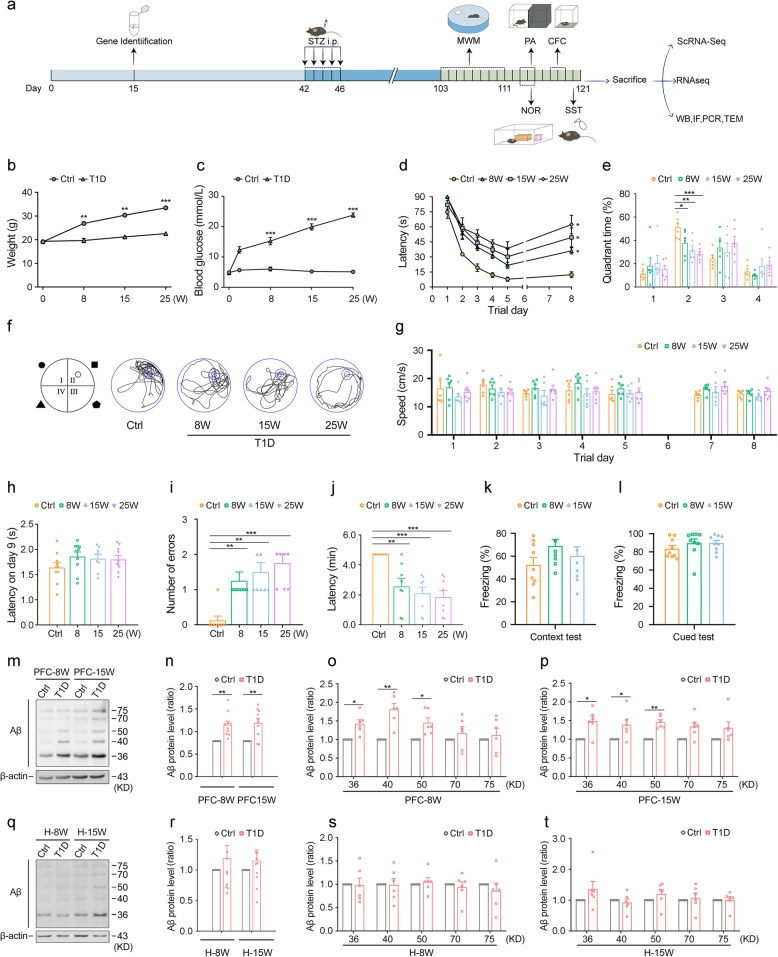
T1D mice display significant memory dysfunction and have increased Aβ levels.**a** Flow chart of animal experiments. **b, c** Body weight (**b**) and blood glucose (**c**) of mice in each group. n = 10. **d** Escape latency of each group for days 1 to 8 by Morris Water Maze (MWM). n = 6. **e, f** Time percent in the target quadrant (**e**) and representative path maps (**f**) of each group following removal of the platform. n = 6. **g** Swimming speeds of each group. n = 6. **h** Escape latency of each group in the MWM visible platform test on day 9. n = 6. **i** Number of errors of each group in passive avoidance (PA) task. n = 8. **j** Latency on day 3 in PA test. n = 8. **k, l **Mean percentage of conditioned freezing in the context test (**k**) and in the cued test of contextual fear conditioning (CFC) (**l**). n = 9. **m-p** Representative images (**m**) and quantitative analysis of Western blot showed the total amount of Aβ oligomers (**n**) and different molecular weight Aβ oligomers in the prefrontal cortex at 8 (**o**) and 15 (**p**) weeks. n = 6. **q-t **Representative images (**q**) and quantitative analysis of Western blot showed the total amount of Aβ oligomers (**r**) and different molecular weight Aβ oligomers in the prefrontal cortex of mice at 8 (**s**) and 15 (**t**) weeks. n = 6. Ctrl: Wild-type nondiabetic group. T1D: Wild-type diabetic group. 8 W, 15 W, 25 W: T1D mice at 8, 15, and 25 weeks post-STZ injection, respectively. PFC-8 W, PFC-15 W: The prefrontal cortex taken from T1D mice at 8 and 15 weeks post-STZ injection, respectively. H-8 W, H-15 W: The hippocampus taken from T1D mice at 8 and 15 weeks post-STZ injection, respectively. Data were presented as mean ± SEM. For in vivo studies, n represents the number of animals per group. For b-e, g-l, One-way ANOVA. For n-p, r-t, two-tailed unpaired t test. * p < 0.05, ** p < 0.01, *** p < 0.001, compared with Ctrl group.

All animal experimental procedures were approved by the Experimental Animal Ethics Committee of Capital Medical University (approval number AEEI-2021-023). A total of 204 male C57BL/6J mice, aged 6–8 weeks, were used in this study. The C57BL/6J mice were purchased from Beijing Vital River Laboratory Animal Technology Co., Ltd., while TREM2 conditional knockout (TREM2 cKO) mice on a C57BL/6J background were obtained from Cyagen Biosciences Inc. The successful knockout of TREM2 gene was confirmed by Western blot (Fig. 5l). The strategy for generating TREM2 conditional knockout mice was schematically illustrated in Figure S6g. The mice were randomly divided into the following groups: wild-type nondiabetic (Ctrl) group, TREM2 cKO group, wild-type T1D group and TREM2 cKO diabetic (T1D + TREM2 cKO) group. The widely used streptozotocin (STZ) model was selected for its reproducibility and capacity to mimic insulin deficiency in T1D [29, 30]. Mice were administered 50 mg/kg STZ intraperitoneally for five consecutive days without insulin treatment as previously described [31]. After two weeks, mice with fasting blood glucose levels exceeding 11.1 mmol/L, as measured in tail vein blood using a handheld glucometer based on the glucose oxidase method, were considered diabetic. Blood glucose levels, weight, and health status were closely monitored. Any animal exhibiting severe distress was humanely euthanized in accordance with animal ethics guidelines. The animals were sacrificed and tissue samples were collected at 8, 15, and 25 weeks following STZ injection. The survival rates of the mice at these time points were 96.3%, 92.3%, and 83.3%, respectively. Figure 1a shows the flow diagram of our animal experiments. Details regarding all chemicals, reagents, antibodies, and instruments used in this study, including their sources and catalog numbers, are provided in Supplementary Tables 1, 2, 3.

### Behavioral tests

In this study, multiple behavioral tests were employed to comprehensively assess the cognitive function of the mice. The morris water maze (MWM) and the novel object recognition (NOR) were chosen to assess hippocampus-dependent spatial memory and prefrontal cortex-dependent declarative memory, respectively. The passive avoidance (PA) task and contextual fear conditioning (CFC) are classic paradigms for assessing associative memory, with their neural circuits involving complex interactions between the prefrontal cortex and the hippocampus [32, 33]. The splash test (SST) was to exclude the potential confounding effects of emotional disorders on cognitive test results.

The MWM was conducted as previously described [31]. Briefly, the mice underwent a 5-day training period, during which the time required to find the fixed hidden platform was recorded as escape latency. On day 7, after removing the platform, the percentage of time that the mice spent in each quadrant was recorded. On day 8, with the hidden platform replaced, the escape latency was recorded again. On day 9, a visible platform test was conducted to assess the sensorimotor abilities and motivation of the mice. Throughout all trials, animals were monitored for signs of excessive distress, which would have resulted in immediate removal. The water temperature was maintained at 22 °C to prevent hypothermia. After each trial, mice were promptly dried with warm towels and returned to their home cages. The data were analyzed using WaterMaze 3 software.

NOR test was carried out as previously described [34]. On day 1, the mice were placed at the center of the empty arena and were allowed a five-minute period for free exploration. On day 2, with two identical objects (A1 and A2) placed in the arena, the total activity and exploration time were recorded while the mice were allowed to explore freely for 10 min or until each object had been explored for at least 20 s. On day 3, the A2 object was replaced with a novel one (b), and exploration was recorded similarly. The recognition index (RI) is calculated using the following formula: RI = (N - F)/(N + F) × 100%. N was the novel object exploration time, and F was the familiar object exploration time.

The experiment of CFC was carried out as previously described [35]. On day 1, mice were introduced to the conditioning chamber for a 3-minute exploration period and received three 30-second tone stimuli, each followed by a 0.6 mA foot shock administered during the last 2 s. On day 2, we conducted context test that mice were returned to the conditioning chamber without any stimuli, and their freezing behavior was observed and recorded for 3 min. On day 3, we conducted cued test that mice were placed in a novel chamber and exposed to the same tone stimulus for 3 min. Freezing behavior was recorded using the Video Freeze^®^ software system.

The PA task was carried out as previously described [36]. On Day 1, mice were placed in the light chamber facing away from the door. After 10 s, mice were allowed to move freely between the two chambers for 3 min. On Day 2, mice entering the dark chamber received a 0.3 mA foot shock, and the session lasted for 5 min. On Day 3, the latency to first enter the dark chamber and the number of entries into the dark chamber (errors) within 5 min were recorded.

The SST test commenced with the application of a 10% sucrose solution to the dorsal fur of the mice in the home cage. Subsequently, the time until the first grooming and the total grooming duration over 5 min were recorded.

### SnRNA-seq

We employed snRNA-seq on 59, 356 cells isolated from the prefrontal cortex and hippocampus of age-matched mice (Ctrl vs. T1D). These regions were selected for this study to minimize the complexity of cell composition and to target brain regions associated with learning and memory. Brain tissue from the hippocampus and prefrontal cortex (approximately 0.01 g and 0.012 g per unilateral structure, respectively) was simultaneously isolated and processed to generate stable cDNA libraries. Cells were loaded into a Chromium Single Cell 3’ Chip and processed in accordance with the manufacturer’s instructions. Library construction was performed using the Chromium Single Cell 3’ Library & Gel Bead Kit v2. The single-cell libraries were sequenced on an Illumina NovaSeq 6000 sequencing system (paired-end multiplexing run, 150 bp) at a minimum depth of 20,000 reads per cell. The 10x Genomics Cell Ranger pipeline was employed for data analysis, according to the manufacturer’s instructions. Samples were integrated and analyzed using Seurat package (V5) in R software (v4.4.0) [37]. The batch effect was adjusted using harmony (v0.1.0) [38]. Genes with significant subpopulation-specific expression (*p* < 0.05), a log FC (Fold change) >0.25 and expressed in at least 20% of the target subpopulation cells, were selected as differentially expressed genes (DEGs). Subpopulation identities were manually annotated based on these DEGs and well-recognized cell markers according to published articles [39]. DEGs in different cell subpopulations were subjected to gene ontology (GO) and Kyoto encyclopedia of genes and genomes (KEGG) analysis, as previously described [40, 41]. Pseudotime trajectory analysis was performed using Monocle2 package (v2.14.0) based on the DDRTree method [42].

### Cell culture

The primary mouse microglia and BV2 cells were prepared as previously described [43, 44]. BV2 cells between passages 6 and 10 were used for all experiments to maintain genetic stability and biological characteristics. Cells were maintained in Dulbecco’s Modified Eagle’s Medium/Nutrient Mixture F-12 (DMEM/F12) supplemented with 10% fetal bovine serum (FBS) and 1% penicillin–streptomycin (100×), and dissociated using 0.25% trypsin. The cell seeding density is shown in Supplementary Table 4.

To establish a high-glucose (HG) condition, a glucose solution was added to the culture medium to a final concentration of 50 mmol/L. After 24 h of HG exposure, cells were transfected with siRNA targeting TREM2 (si-TREM2) for 6 h, or transduced with TREM2-overexpressing adenovirus (TREM2-OE) for 12 h. The efficiency of TREM2 overexpression was confirmed by Western blot (Fig. 7g). A schematic of the experimental timeline is shown in Fig. 6a.

### Wound scratch and transwell assays

For the wound scratch assay, linear scratch wounds were created on the BV2 cell monolayer. Images of the scratch were captured at 0 h as the baseline. Images were continued to be captured at 12, 24, and 48 h to monitor cell migration into the scratch area [45]. For the transwell assay [45], 100 µM Aβ oligomers (oAβ) or 100 µM Aβ fibrils (fAβ) was added to the lower chamber and incubated for 48 h to observe BV2 cell migration. After 48 h of culture, the cells on top of the filter membrane were removed, and those beneath were stained using crystal violet. The time points for the wound scratch assay and Transwell assay in BV2 cells were detailed in Fig. 6a.

### Flow cytometry and living cell imaging

For the flow cytometry, BV2 cells were treated with fluorescently-labeled Aβ at a final concentration of 0.5 μM for 2 hours. Subsequently, the proportion of Aβ-positive cells was measured using a flow cytometer, which reflected the phagocytic activity of the BV2 cells.

Live-cell imaging was performed on BV2 cells treated with fluorescently labeled Aβ (at a final concentration of 0.5 µM) for 2 h, with confocal images captured at 2-min intervals.The fluorescence intensity of Aβ within BV2 cells was analyzed using Image J software. The time points for the flow cytometry and living cell imaging in BV2 cells were detailed in Fig. 6a.

### ROS assay

Intracellular ROS levels were measured using a commercial 2’,7’-dichlorodihydrofluorescein diacetate (DCFH-DA) assay kit following the manufacturer’s protocol. Briefly, BV2 cells grown on glass coverslips in 24-well plates were incubated with 10 µM DCFH-DA in serum-free medium at 37 °C for 20 min in the dark. Following three gentle washes with warm PBS to remove excess probe, the cells were imaged using a laser scanning confocal microscope to quantify the green fluorescence intensity.

### Western blot

The Western blot experiment was carried out as previously described [31]. The signals were detected using a chemiluminescence method and quantified using the Fusion FX6 XT imaging system with Fusion Capt 16.15 software. Protein levels were quantified by densitometry and normalized to β-actin, α-tubulin, or GAPDH, which served as the internal standard.

### Immunofluorescence

The immunofluorescence experiment was conducted as previously described [46]. For each mouse, five consecutive Sect. (20 μm thickness each) were collected from the prefrontal cortex (Fig. 4c) and hippocampus (Fig. 4j). To ensure consistency in image quantification, all imaging was performed under identical acquisition parameters (Supplementary Table 5). Images were acquired using a laser scanning confocal microscope and quantified with ImageJ software. For statistical comparison across groups, the data (fluorescence intensity, raw cell count, or fluorescence area) from each experimental group were normalized to the corresponding mean values of the control group, yielding relative values. These normalized data were subjected to subsequent statistical analysis.

### Enzyme-linked immunosorbent assay (ELISA)

The supernatants from the prefrontal cortex of mice were collected, and the levels of Aβ1–42 were measured using the Mouse Aβ1–42 ELISA kit according to the manufacturer’s protocol. Each sample was assayed in three replicates to ensure reliability of the measurements.

### Quantitative real-time polymerase chain reaction (qRT-PCR)

The qRT-PCR was carried out as previously described [46]. The reaction protocol consisted of an initial denaturation at 95 °C for 30 s (1 cycle), followed by 40 cycles of denaturation at 95 °C for 5 s and annealing/extension at 60 °C for 30 s. A melting curve analysis was subsequently performed to confirm amplification specificity. Primer sequences were designed and sourced as follows: primers for TREM2, Cst7, and Cxcl10 were designed using the NCBI Primer-BLAST tool (https://www.ncbi.nlm.nih.gov/tools/primer-blast/). The sequences were as follows: TREM2: forward, 5’–GGAGGACCCTCTAGATGACCAAGA–3’; reverse, 5’–AGGCCAGGAGGAGAAGAATGGA–3’. Cst7: forward, 5’–GGAGCTGTACTTGCCGAGC–3’; reverse, 5’–CATGGGTGTCAGAAGTTAGGC–3’. Cxcl10: forward, 5’–CCAAGTGCTGCCGTCATTTTC–3’; reverse, 5’–GGCTCGCAGGGATGATTTCAA–3’. Primer sequences for GAPDH [47], Lpl [48], Ccr5 [49], and Cx3cr1 [50] were obtained from previous reports. Conventional PCR was carried out on a MyCycler thermal cycler, and quantitative real-time PCR was performed using a QuantStudio 5 system. Relative gene expression was calculated using the 2^(–ΔΔCt) method [46].

### Bulk RNA sequencing (RNA-Seq)

We performed RNA-seq analysis on the prefrontal cortex at 8 weeks after STZ injection. The DEseq2 R package was utilized for differential analysis. DEGs were identified based on the criteria of *p* < 0.05 and an absolute value of |log_2_FC| >1. R packages of ggplot2 and ComplexHeatmap were implemented to visualize the data with volcano maps and heat maps. The DEGs were uploaded to the STRING database (version 11.5, available at https://cn.string-db.org/) to construct a protein-protein interaction (PPI) network, which was subsequently visualized using Cytoscape 3.9.0. For GO and KEGG pathway analysis, the DEGs were analyzed using DIANA Tools (http://www.microrna.gr/), with significance assigned to pathways (*p* < 0.05). Finally, Gene Set Enrichment Analysis (GSEA) was performed using the clusterProfiler R package (v4.0) to identify significantly enriched Hallmark gene sets from MSigDB (https://www.gsea-msigdb.org/gsea/msigdb/index.jsp).

### Statistical analysis

Data were presented as means ± standard error of the mean (SEM) and were analyzed using GraphPad Prism (San Diego, CA, USA). To minimize bias, animals were randomly assigned to experimental groups, and the investigators were blinded to group allocation during the experiment and when assessing the outcomes. The normality of data distribution was assessed using the Shapiro-Wilk test, and the homogeneity of variances was verified using Brown-Forsythe test. For comparisons between two groups, a two-tail unpaired Student’s t test was used. For multiple comparisons, one-way analysis of variance (ANOVA) was performed, followed by Tukey’s post hoc test for normally distributed data or an appropriate non-parametric alternative if the data did not meet the assumptions of normality or equal variance. Values of *p* < 0.05 were considered statistically significant.

## Result

### T1D mice display significant memory dysfunction

T1D mice at 8 weeks after STZ injection showed a significant decrease in body weight (*p* < 0.0001, Fig. 1b) and an increase in blood glucose levels compared to Ctrl mice (*p* < 0.0001, Fig. 1c). Meanwhile, food and water intake, as well as urine output, were increased in T1D mice (data not shown). The MWM test was performed to assess the learning and memory defects in T1D mice. Compared to Ctrl mice, T1D mice had significantly longer escape latency (Ctrl vs. 8 W: *p* = 0.0002, Ctrl vs. 15 W: *p* = 0.0038, Ctrl vs. 25 W: *p* = 0.0004, Fig. 1d), significantly less time in the target quadrant (Ctrl vs. 8 W: *p* = 0.0471, Ctrl vs. 15 W: *p* = 0.0007, Ctrl vs. 25 W: *p* = 0.0001, Fig. 1e), and more chaotic swimming paths (Fig. 1f). As time progressed, T1D mice at 15 and 25 weeks after STZ injection exhibited worsening learning and memory abilities (Fig. 1d-f). There was no significant difference in swimming speed among the mice on each day (Fig. 1g). To determine whether there was a difference in visual acuity among the mice, a visible-platform test was conducted on the 9th day of MWM, and no significant differences were observed among the groups (Fig. 1h). The results of PA task revealed significantly higher number of errors (Ctrl vs. 8 W: p = 0.0091, Ctrl vs. 15 W: p = 0.0012, Ctrl vs. 25 W: p = 0.0002, Fig. 1i) and shorter latency in T1D mice (Ctrl vs. 8 W: *p* = 0.0091, Ctrl vs. 15 W: *p* = 0.0012, Ctrl vs. 25 W: *p* = 0.0002, Fig. 1j), with the discrepancy increasing as the duration of the disease prolonged (Fig. 1i, j). The results of the CFC test showed no observable difference in freezing time of context test and cued test among groups (Fig. 1k, l), indicating that T1D had minimal effect on fear memory in mice. In summary, T1D mice display significant learning and memory impairments, and the degree of cognitive dysfunction increases with the duration of the disease.

### The level of Aβ increases in the prefrontal cortex of T1D mice

Western blot results showed that the total expression of Aβ oligomers in the prefrontal cortex was dramatically increased in T1D mice at 8 and 15 weeks after STZ injection, particularly for the 36 kDa, 40 kDa and 50 kDa Aβ oligomers compared to Ctrl mice (Fig. 1n: PFC-8 W: *p* = 0.001, PFC-15 W: *p* = 0.0006; Fig. 1o: 36KD: *p* = 0.0412, 40KD: *p* < 0.0001, 50KD: *p* = 0.0258; Fig. 1p: 36KD: *p* = 0.0027, 40KD: *p* = 0.0235, 50KD: *p* = 0.0050, Fig. 1m-p). A trend toward increased Aβ oligomers was observed in the hippocampus of T1D mice, although it did not reach statistical significance (Fig. 1q-t). At 8 weeks after STZ injection, no obvious Aβ (6E10 labeled) plaques were observed in the prefrontal cortex of T1D mice (sFig. 5e). This absence of plaque deposition persisted even when the observation period was extended to 25 weeks (data not shown). However, at this late stage, a subset of T1D mice exhibited severe health deterioration, including metabolic disturbances. Out of ethical considerations for animal welfare and to ensure the reliability of molecular data from stable physiological conditions, subsequent in-depth mechanistic investigations were primarily focused on the 8-week time points.

In summary, these results suggest that early cognitive and behavioral changes in the T1D mice may be associated with the accumulation of Aβ oligomers, rather than being directly linked to the formation of mature Aβ plaques. Particularly in the prefrontal cortex, the pathological increase in Aβ oligomers appears to occur relatively early.

### Single-nucleus analysis of cell types and microglial subpopulations in T1D brain

**Fig. 2:**
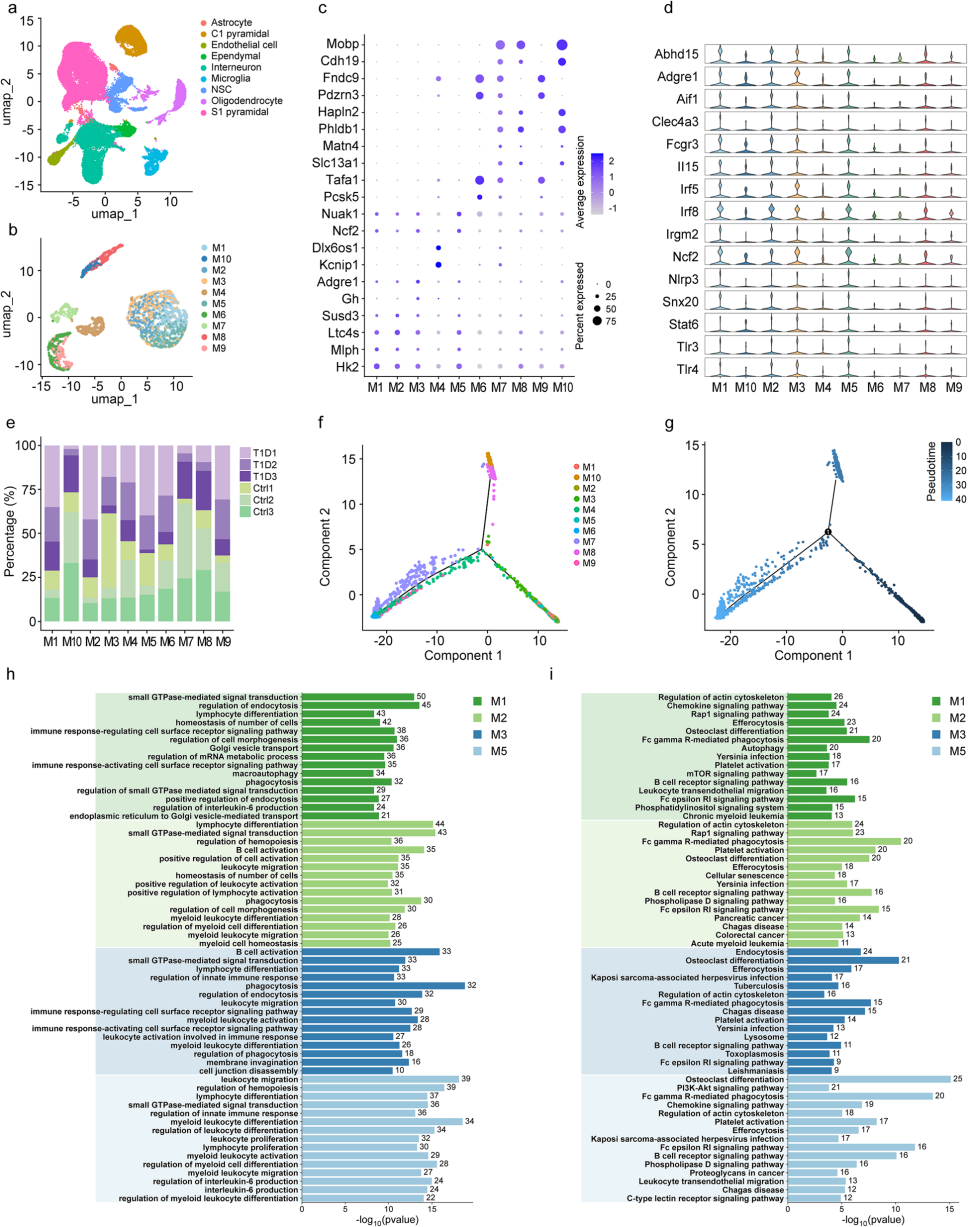
Characterization and functional analysis of microglia subpopulations in T1D mice. **a** Unbiased clustering of 59,356 cells across six samples. **b** UMAP plot showing the microglia subpopulations. **c** Bubble plot showing the expression of the established marker genes for each microglia subpopulation. **d** Violin plots showing the expression of feature functional genes in each microglia subpopulation. **e** Relative proportion of each sample in the each microglia subpopulation. **f** Pseudotime analysis of microglial subpopulations revealed subpopulation-specific trajectories in gene expression. **g** Pseudotime analysis of microglia, colored by pseudotime, illustrated the dynamics of gene expression across pseudotime. **h** GO terms enrichment in subpopulations of M1, M2, M3 and M5. **i** KEGG pathways enrichment in subpopulations of M1, M2, M3 and M5. M: Microglia, Ctrl: Wild-type nondiabetic group. T1D: Wild-type diabetic group. GO: Gene ontology, KEGG: Kyoto encyclopedia of genes and genomes. *n* = 3. For in vivo studies, n represents the number of animals per group

We conducted snRNA-Seq in the prefrontal cortex and hippocampus of T1D and Ctrl mice, identifying cell types and capturing a total of 59,356 cells. All major cell types of the central nervous system (CNS), including neurons, astrocytes, oligodendrocytes, microglia, endothelial cells, ependymal cells and neural stem cells (Fig. 2a), were observed. We identified ten distinct microglia subpopulations, seven distinct neuronal subpopulations, five distinct oligodendrocyte subpopulations, and three distinct astrocyte subpopulations (Fig. 2b, Fig. S1a, S2a, S3a). Bubble plots displayed the expression of common markers for neurons, oligodendrocytes and astrocytes (Fig. S1b, S2b, S3b). GO analysis of neuronal, astrocyte, and oligodendrocyte subpopulations was shown in Figures S1c-i, S2c-e, and S3c-g, respectively. 

Microglia (total 3,208 cells) were clustered and identified into 10 functionally specialized subpopulations (M1-M10) (Fig. 2b). Bubble plots and heatmaps demonstrated the specific marker genes for each subpopulation (e.g., Mclop for M1, Cdh19 for M3, Fndc9 for M5, etc.) (Fig. 2c, Supplementary Fig. S4a). The expression intensity of functionally relevant marker genes (e.g., Slc13a1, Nuak1, Kcnp1) exhibited significant subpopulation-specific differences in violin plots, with M1, M2, M3, and M5 subpopulations showing high expression of genes related to phagocytosis, immune regulation, and inflammation (Fig. 2d, Supplementary Fig. S4c). Significant differences in subpopulation proportions were observed between the T1D and Ctrl groups (Fig. 2e, Supplementary Fig. S4b), with M1, M2, and M5 subpopulations markedly increased in the T1D group compared to the Ctrl group. Pseudotime trajectory analysis revealed that microglia transitioned from M1, M2, M3, and M5 toward M8, M10 and M4, M6, M7, M9 along the temporal axis (Fig. 2f), suggesting that T1D may influence neuropathological progression by altering microglial functional states.

To investigate the functional heterogeneity of each microglial subpopulation, we performed GO biological process enrichment analysis and KEGG pathway enrichment analysis for each subpopulation (Fig. 2h-i, Fig. S4d-i, Supplementary Table 6). Our GO enrichment analysis identified that the M1, M2, M3, and M5 subpopulations of microglia shared involvement in two critical biological processes: small GTPase-mediated signal transduction and lymphocyte differentiation. Small GTPase-mediated signal transduction is a fundamental pathway that regulates a variety of cellular functions, including cytoskeletal dynamics, vesicle trafficking, and cell migration (Fig. 2h-i, Fig. S4j). The shared involvement of these subpopulations in this pathway suggests a coordinated role in modulating immune responses and maintaining cellular homeostasis. Lymphocyte differentiation, on the other hand, highlights the potential for crosstalk between microglia and the adaptive immune system, which might be crucial for immune surveillance and the resolution of inflammation in the CNS. Further, our KEGG pathway enrichment analysis revealed that the M1, M2, M3, and M5 subpopulations shared seven signaling pathways, which are closely related to the biological processes identified in the GO analysis. These shared pathways include the regulation of the actin cytoskeleton, efferocytosis, osteoclast differentiation, Fc gamma R-mediated phagocytosis, platelet activation, B cell receptor signaling pathway, and Fc epsilon RI signaling pathway (Fig. 2h-i, Fig. S4k). Notably, pathways such as the regulation of the actin cytoskeleton and Fc gamma R-mediated phagocytosis are directly linked to the small GTPase-mediated signal transduction identified in the GO analysis, underscoring the importance of these pathways in microglial function. KEGG pathway enrichment analyses for the other subpopulations could be found in Fig. S4d-i.

While these subpopulations exhibited shared functional profiles, they also displayed distinct functional signatures that underscored their specialized roles in immune regulation and cellular processes. The M1 subpopulation participated in immune activation functions (including immune response signaling and IL-6 regulation), metabolic regulation (endocytosis, mRNA metabolic process control, macroautophagy), vesicular transport systems (Golgi vesicle transport), cell morphogenesis modulation, and phagocytosis. The M2 subpopulation was enriched in hematopoietic regulation, immune cell activation (B cells, lymphocytes, and leukocytes), myeloid leukocyte differentiation and homeostasis, leukocyte migration, and phagocytosis. The M3 subpopulation was associated with innate immune response regulation, dynamic phagocytic processes (phagocytosis, endocytosis, and membrane invagination), leukocyte migration, myeloid leukocyte activation, cell junction disassembly, and B cell activation. The M5 subpopulation was involved in leukocyte migration and proliferation (both lymphoid and myeloid lineages), hematopoietic regulation, myeloid differentiation control, innate immune response modulation, and interleukin-6 production pathways.

**Fig. 3:**
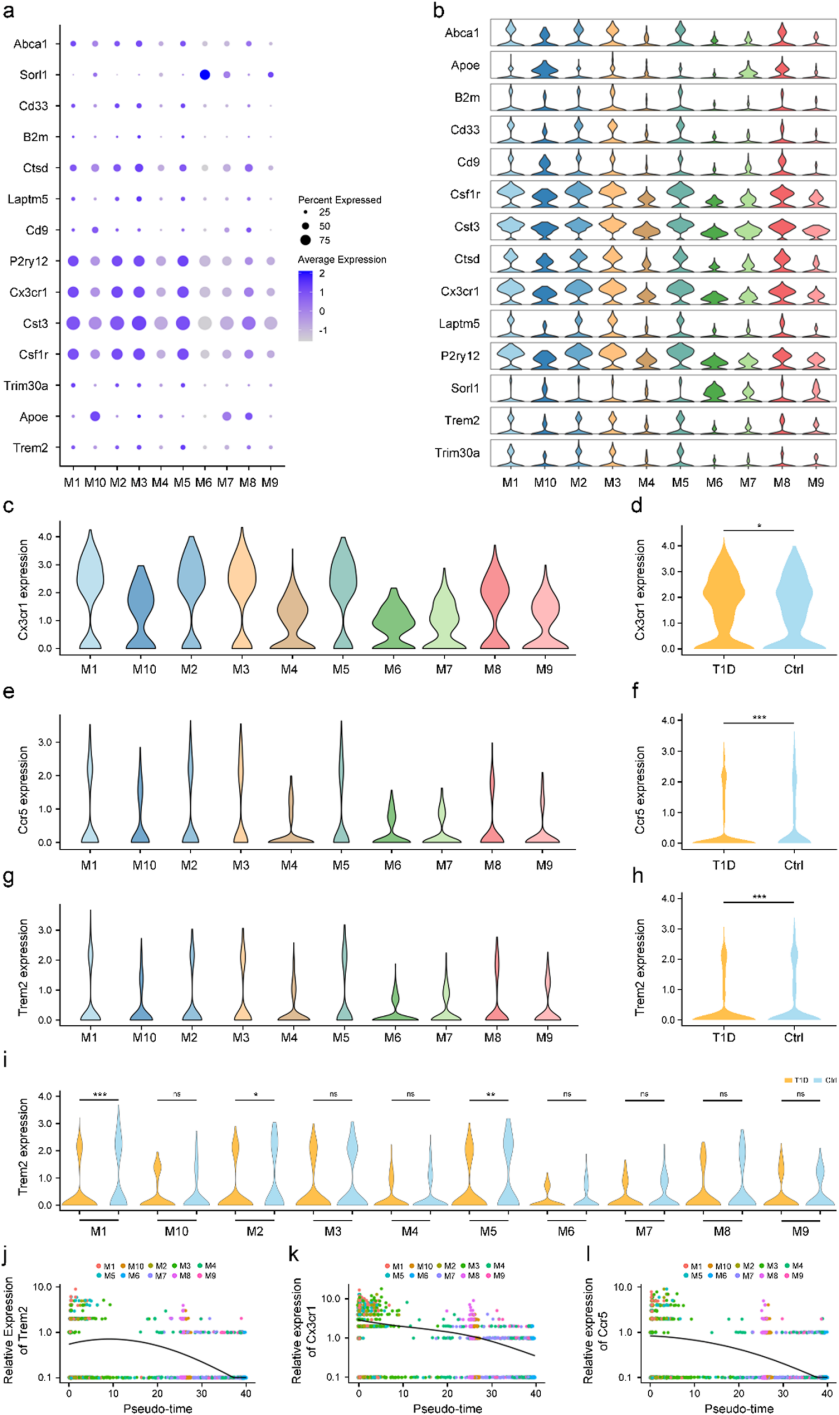
Distribution and expression dynamics of Aβ-related genes in microglial subpopulations of T1D mice. **a, b** Bubble plot (**a**) and violin plots (**b**) showing the expression of Aβ-related genes for each microglia subpopulation. **c**,** e**,** g** Violin plots showing the expression of Cx3cr1 (**c**), Ccr5 (**e**) and Trem2 (**g**) in the microglial subpopulations. **d**,** f**,** h** Violin plots comparing the levels of Cx3cr1 (**d**), Ccr5 (**f**) and Trem2 (**h**) in microglia between the Ctrl and T1D groups. **i** Violin plot showing the levels of Trem2 among microglia subpopulations. **j-l** Smoothed expression curves of Trem2 (**j**), Cx3cr1 (**k**) and Ccr5 (**l**) along the trajectory in microglia subpopulations. M: Microglia, Ctrl: Wild-type nondiabetic group. T1D: Wild-type diabetic group. *n* = 3. For in vivo studies, n represents the number of animals per group. For c-i, unpaired t test. * *p* < 0.05, ** *p* < 0.01, *** *p* < 0.001, compared with Ctrl group

We further analyzed the expression patterns of Aβ phagocytosis-related genes across these subpopulations. The results showed significant differences in the expression of these genes among subpopulations, with Aβ phagocytosis-related genes highly expressed in M1, M2, M3, and M5 subpopulations (Fig. 3a, b). Notably, Trem2, Cx3cr1, and Ccr5 were highly expressed in M1, M2, M3, and M5 subpopulations (Fig. 3c, e, g) and showed significantly higher expression in the T1D group compared to the Ctrl group, suggesting these genes may play critical roles in T1D pathology (Trem2: *p* < 0.001; Cx3cr1: *p* < 0.05; Ccr5: *p* < 0.001, Fig. 3d, f, h). Further analysis revealed that Trem2 expression was significantly higher in the M1, M2, and M5 subpopulations of the T1D group compared to the Ctrl group (M1: *p* < 0.001; M2: *p* < 0.05; M5: *p* < 0.01, Fig. 3i).

Pseudotime trajectory analysis was performed to explore the dynamic expression trends of these genes during microglial differentiation. The results showed high expression of Trem2 in M1, M2, M3, and M5 subpopulations, while Cx3cr1 and Ccr5 were highly expressed in M3 and M5 subpopulations. However, the expression of all three genes gradually decreased over time (Fig. 3j), suggesting their potential roles in regulating microglial functional and activation states.

### Microglia activation in the prefrontal cortex and hippocampus of T1D mice

**Fig. 4:**
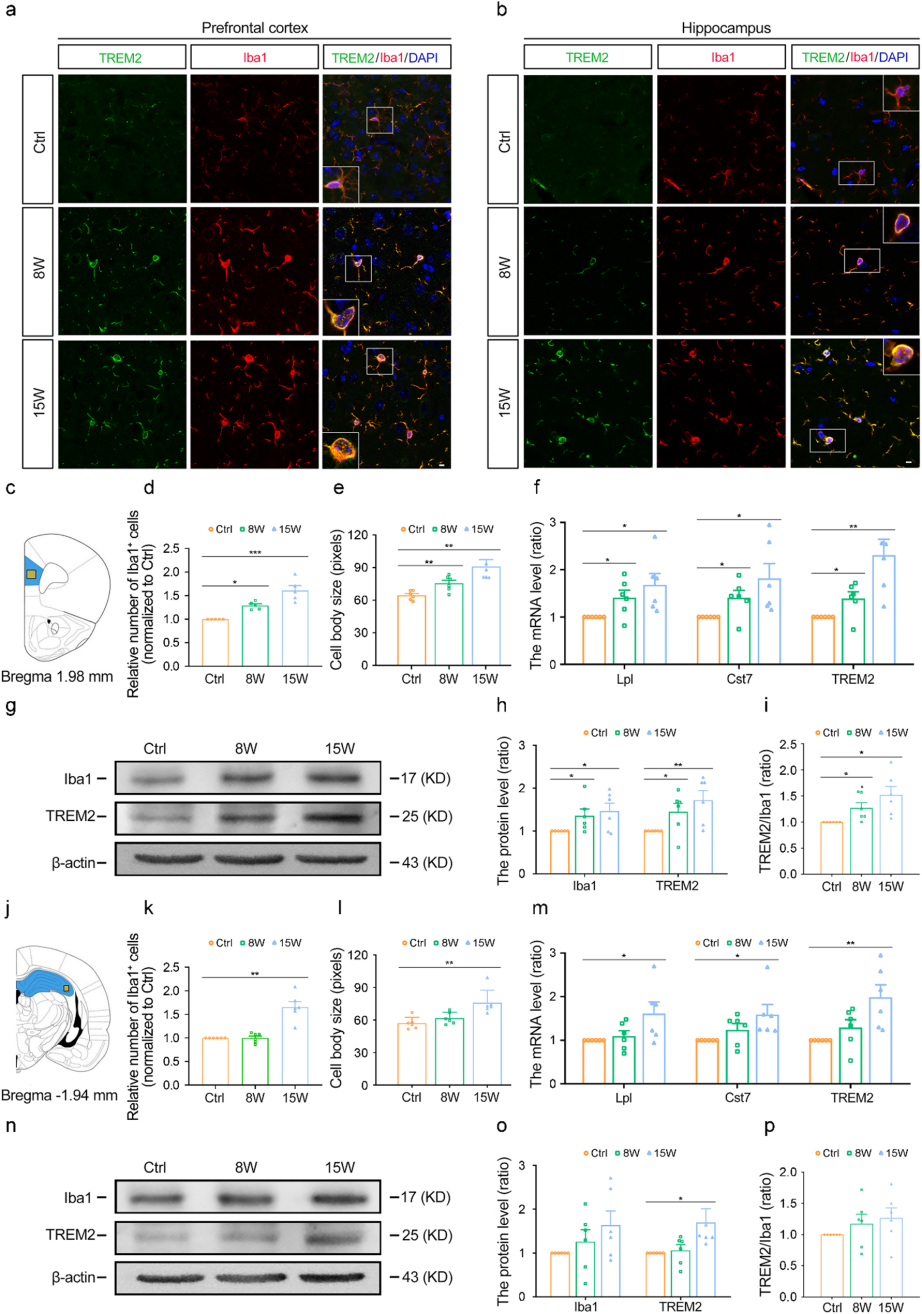
Microglial activation and TREM2 expression in the prefrontal cortex and hippocampus of T1D mice. **a**,** b** Representative images of immunofluorescent staining of Iba1 (red), TREM2 (green) and DAPI (blue) in the prefrontal cortex (**a**) and hippocampus (**b**). Scale bar = 5 μm. **c**,** j **Schematic diagrams of prefrontal cortex (**c**) and hippocampus (**j**). **d** The relative number of Iba1-positive cells in the prefrontal cortex normalized to the control group. **e** The soma size of Iba1-positive cells in the prefrontal cortex. **f**,** m** The relative mRNA levels of LPL, CST7 and TREM2 in the prefrontal cortex (**f**) and hippocampus (**m**). *n* = 6. **g**,** h**,** i** Representative images (**g**) and quantitative analysis of Western blot showed the protein levels of Iba1 and TREM2 (**h**), and the TREM2/Iba1 ratio (**i**) in the prefrontal cortex. *n* = 6. **k** The relative number of Iba1-positive cells in the hippocampus normalized to the control group. *n* = 6. **l** The soma size of Iba1-positive cells in the hippocampus. *n* = 6. **n**,** o**,** p** Representative images (**n**) and quantitative analysis of Western blot showed the protein levels of Iba1 and TREM2 (**o**), and the TREM2/Iba1 ratio (**p**) in the hippocampus. *n* = 6. Ctrl: Wild-type nondiabetic group. 8 W, 15 W: T1D mice at 8 and 15 weeks post-STZ injection, respectively. Data were presented as mean ± SEM. For in vivo studies, n represents the number of animals per group. For d-f, h-i, k-m, o-p, One-way ANOVA. * *p* < 0.05, ** *p* < 0.01, *** *p* < 0.001, compared with Ctrl group.

After labeling microglia with Iba1, we observed a resting morphology in microglia, characterized by small cell bodies and thin, elongated processes in the prefrontal cortex and hippocampus of Ctrl mice (Fig. 4a, b). In the T1D group, the number of microglia in the prefrontal cortex significantly increased at 8 weeks after STZ injection, exhibiting larger cellular bodies, this effect became even more pronounced at 15 weeks (Fig. 4d: Ctrl vs. 8 W: *p* = 0.0194, Ctrl vs. 15 W: *p* < 0.0001; Fig. 4e: Ctrl vs. 8 W: *p* = 0.0081, Ctrl vs. 15 W: *p* = 0.0024, Fig. 4a, d and e). At 8 weeks, the number of microglia in the hippocampus did not change significantly, and there was no significant difference in the size of cell bodies between Ctrl and T1D groups (Fig. 4b, k and l). At 15 weeks, the number of microglia in the hippocampus significantly increased, exhibiting an active morphology with enlarged cell bodies (Fig. 4k: Ctrl vs. 15 W: *p* < 0.0001; Fig. 4l: Ctrl vs. 15 W: *p* = 0.0030, Fig. 4b, k and l). Similarly, Western blot results revealed that the expression of Iba1 in the prefrontal cortex of the T1D group significantly increased at 8 weeks, and even more so at 15 weeks (Ctrl vs. 8 W: *p* = 0.0468, Ctrl vs. 15 W: *p* = 0.0307 , Fig. 4g, h). At 8 and 15 weeks after STZ injection, Iba1 expression in the hippocampus showed an increasing trend, although it was not statistically significant (Fig. 4n, o). Overall, these results suggest that microglial activation in the prefrontal cortex of T1D mice occurs earlier than in the hippocampus, aligning with the observed changes in Aβ oligomer levels in the brain.

### Expression dynamics of TREM2 in the prefrontal cortex and hippocampus of T1D mice

TREM2, LPL, and CST7 are crucial regulators of microglial phagocytosis, functioning through distinct yet complementary mechanisms. TREM2, a key receptor on microglia, initiates phagocytic signaling. LPL supports this process by enhancing lipid metabolism to fuel ATP production, thereby promoting the clearance of amyloid deposits [51]. By localizing to the endolysosomal compartment and inhibiting cysteine proteases, CST7 thereby regulates proteolytic activity, which is essential for modulating phagocytic function [52]. In T1D mice, the mRNA levels of TREM2, LPL and CST7 in the prefrontal cortex significantly increased at 8 weeks after STZ injection and further increased at 15 weeks (LPL: Ctrl vs. 8 W: *p* = 0.0266, Ctrl vs. 15 W: *p* = 0.0171; CST7: Ctrl vs. 8 W: *p* = 0.0241, Ctrl vs. 15 W: *p* = 0.0258; TREM2: Ctrl vs. 8 W: *p* = 0.0211, Ctrl vs. 15 W: *p* = 0.0029, Fig. 4f). Among these genes, the upregulation of TREM2 was particularly notable. Although the mRNA levels of TREM2, LPL and CST7 in the hippocampus of T1D mice remained unchanged at 8 weeks after STZ injection, a significant increase was observed at 15 weeks, particularly for TREM2 (TREM2: Ctrl vs. 15 W: *p* = 0.0075, Fig. 4m). The TREM2/Iba1 ratio, which reflects the level of TREM2 in individual microglia, was significantly higher in the prefrontal cortex of the T1D group compared to the Ctrl group at both 8 and 15 weeks (Ctrl vs. 8 W: *p* = 0.0242, Ctrl vs. 15 W: *p* = 0.0107, Fig. 4i). In contrast, in the hippocampus, the increase was not statistically significant (Fig. 4p). The expression of TREM2 in the prefrontal cortex and hippocampus of T1D mice was consistent with the alterations in Aβ oligomers, indicating that TREM2 may be involved in the clearance of Aβ oligomers.

### TREM2 deletion exacerbates cognitive impairment in T1D mice

**Fig. 5:**
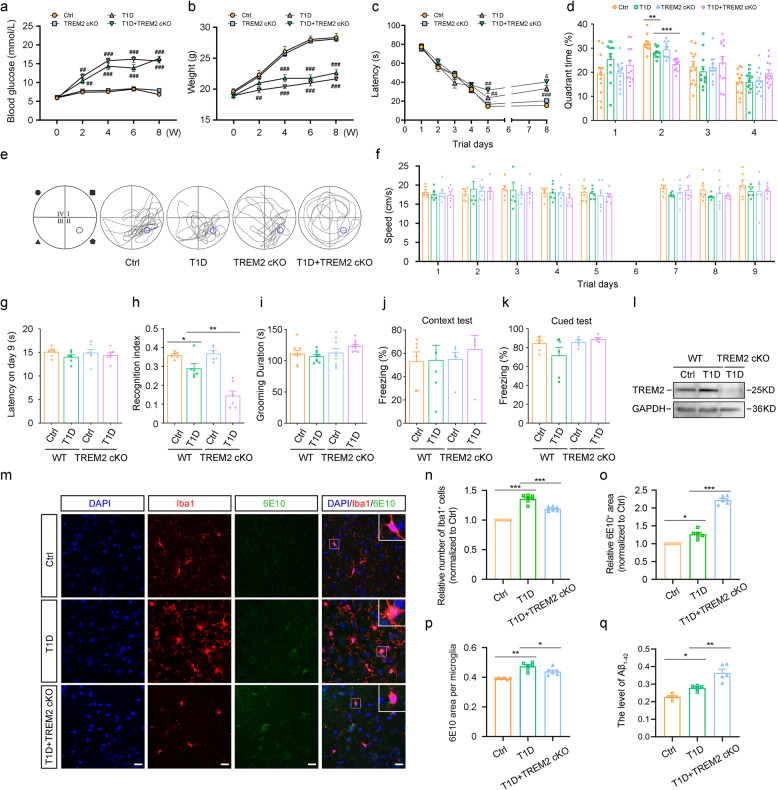
TREM2 deletion aggravates cognitive impairment and promotes Aβ accumulation in the prefrontal cortex of T1D mice. **a**,** b** Blood glucose (**a**) and body weight (**b**) of mice in each group. *n* = 13. **c** Escape latency of each group for days 1 to 8 by Morris water maze (MWM). *n* = 12. **d**,** e** Time percent in the target quadrant (**d**) and representative path maps (**e**) of each group following removal of the platform. *n* = 12. **f **Swimming speeds of each group. *n* = 6. **g** Escape latency of each group in the MWM visible platform test on day 9. *n* = 7. **h** Recognition index in the novel object recognition test. *n* = 6. **i)** Grooming duration in the splash test. *n* = 9. **j**,** k** Mean percentage of conditioned freezing in the context test (**j**) and in the cued test (**k**). Ctrl, *n* = 6; T1D, *n* = 6; TREM2 cKO, *n* = 6 and T1D + TREM2 cKO, *n* = 5. **l** Representative images of Western blot showed the TREM2 expression in the prefrontal cortex. **m** Representative images of immunofluorescent staining of Iba1 (red), 6E10 (green) and DAPI (blue) in the prefrontal cortex. Scale bar = 20 μm. **n**,** o**,** p** Relative number of Iba1 positive cells (n), relative fluorescent area of 6E10 (**o**) and percentage of 6E10 area per micoglia (**p**) in the prefrontal cortex. *n* = 6. **q** The level of Aβ1–42 in the prefrontal cortex by ELISA. *n* = 5. WT: Wild-type. TREM2 cKO: TREM2 knockout. Ctrl: Wild-type nondiabetic group. T1D: Wild-type diabetic group. T1D + TREM2 cKO: TREM2 knockout diabetic group. Data were presented as mean ± SEM. For in vivo studies, n represents the number of animals per group. For a-d, f-k, n-q, One-way ANOVA. * p < 0.05, ** p < 0.01, *** p < 0.001. ## p < 0.01, ### p < 0.001, compared with Ctrl group. & p < 0.05, compared with T1D group.

In this study, we generated mice with a microglia-specific deletion of TREM2. The deletion of TREM2 did not affect the body weight or blood glucose levels of the mice (Fig. 5a, b). The results of the MWM indicated that there were no significant differences between the Ctrl group and the TREM2 cKO nondiabetic group. However, compared to the wild-type T1D mice, the T1D + TREM2 cKO mice exhibited a longer latency to locate the platform (Day 8: Ctrl vs. T1D: *p* < 0.0001, T1D vs. T1D + TREM2 cKO: *p* = 0.0258, Fig. 5c) and spent less time in the target quadrant (Second quadrant: Ctrl vs. T1D: *p* = 0.0113, T1D vs. T1D + TREM2 cKO: *p* = 0.0114, Fig. 5d). Additionally, T1D + TREM2 cKO mice displayed more disorganized swimming paths (Fig. 5e). The speed and visible platform latency showed no difference among groups (Fig. 5f, g). In the NOR test, no significant differences were observed between the Ctrl and the TREM2 cKO groups. However, the T1D + TREM2 cKO mice showed a significant reduction in the recognition index compared to the wild-type T1D mice (Ctrl vs. T1D: *p* = 0.0243, T1D vs. T1D + TREM2 cKO: *p* < 0.0001, Fig. 5h). In the SST, as well as in the contextual and cued fearing conditioning tests of the CFC test, the T1D + TREM2 cKO mice did not display significant differences compared to the other groups (Fig. 5i-k). These results indicate that TREM2 deletion does not affect fear memory or depressive-like behaviors but further exacerbates the cognitive impairments associated with T1D.

### TREM2 deletion promotes Aβ accumulation in the prefrontal cortex of T1D mice

Compared to Ctrl mice, T1D mice exhibited a notable increase in Iba1-positive microglia, 6E10 fluorescence area, and 6E10 expression in microglia of the prefrontal cortex (Fig. 5m-p). In contrast, TREM2 deletion in T1D mice significantly reduced the number of microglia, increased the 6E10 fluorescence area, and decreased 6E10 expression in microglia (Fig. 5n: Ctrl vs. T1D: *p* < 0.0001, T1D vs. T1D + TREM2 cKO: *p* = 0.0249; Fig. 5o Ctrl vs. T1D: *p* = 0.0004, T1D vs. T1D + TREM2 cKO: *p* < 0.0001; Fig. 5p: Ctrl vs. T1D: *p* = 0.0243, T1D vs. T1D + TREM2 cKO: *p* < 0.0001, Fig. 5m-p). ELISA results revealed that the Aβ1–42 level in the prefrontal cortex of the T1D + TREM2 cKO group was significantly elevated compared to that of the wild-type T1D group (Ctrl vs. T1D: *p* = 0.0460, T1D vs. T1D + TREM2 cKO: *p* = 0.0021, Fig. 5q). These data suggest that TREM2 deletion decreases microglial numbers and enhances Aβ levels in the prefrontal cortex of T1D mice.

### Impact of TREM2 on microglial migration under high glucose condition

Immunofluorescence was utilized to detect the co-localization of Iba1, MAP2 and 6E10. Compared to Ctrl mice, microglia showed a heightened propensity to migrate towards 6E10-positive neurons in the frontal cortex of wild-type T1D mice. However, we noted an increased distance between microglia and 6E10-positive neurons in the brains of T1D + TREM2 cKO mice (Fig. S5e).

High glucose treatment for 6 and 12 h slightly increased, while 24 h significantly elevated TREM2 levels in BV2 cells (*p* = 0.0125, Fig. S5a, b). Immunofluorescence indeed revealed a significant increase in TREM2 expression at 24 h (*p* < 0.0001, Fig. S5c, d). Consequently, a 24-hour high glucose treatment was chosen for subsequent experiments.

**Fig. 6:**
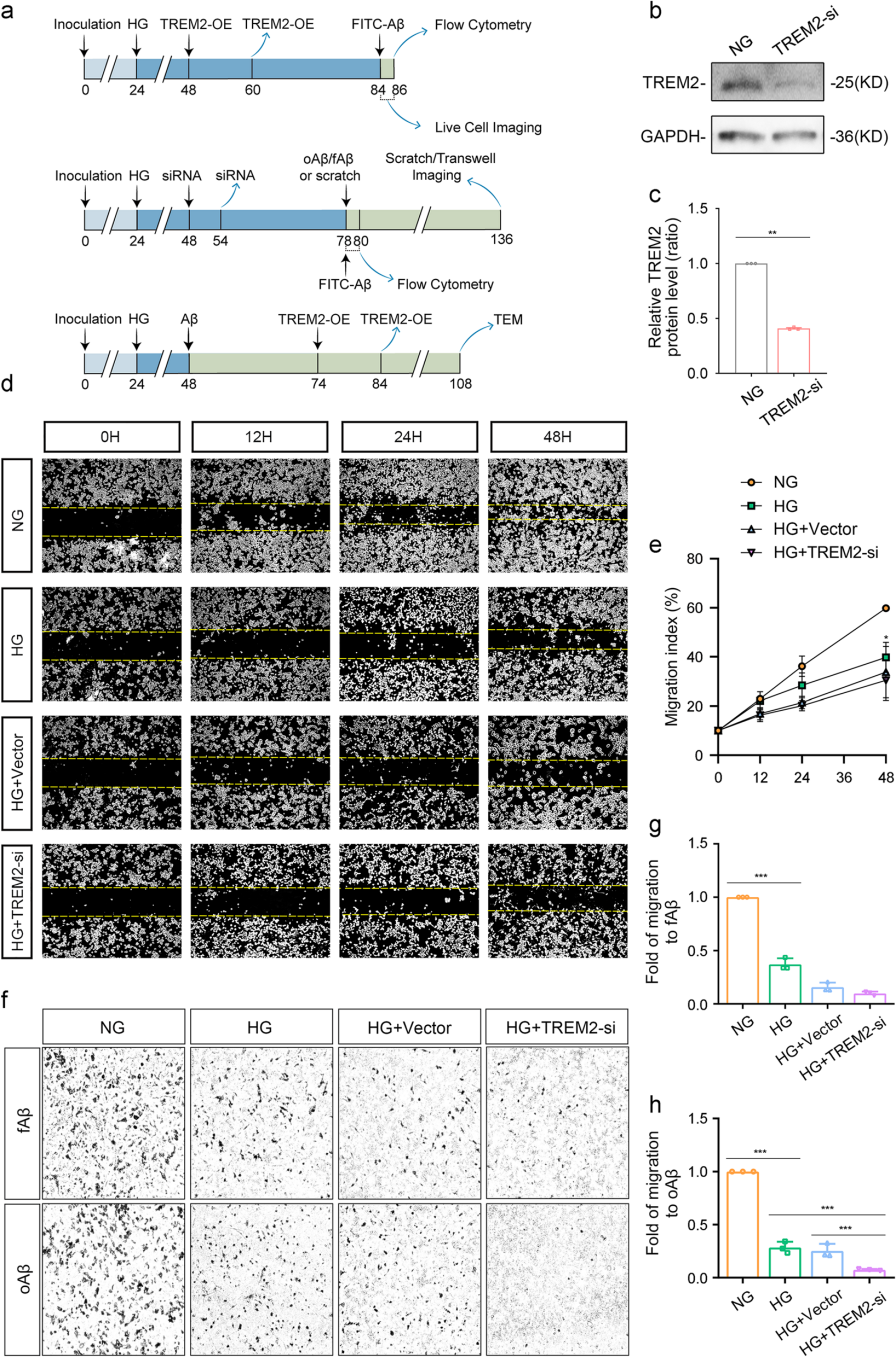
The impact of TREM2 on microglial cell migration towards different forms of Aβ. **a** Flow chart of cell experiments. **b**,** c** Representative images (**b**) and quantitative analysis of western blot (**c**) showed the expression of TREM2 in BV2 cells. *n* = 3. **d**,** e** Representative images (**d**) and migration index of BV2 cells (**e**) at different time points post scratch. *n* = 3. **f** Representative images of cell migration in BV2 cells analyzed by Transwell assay. **g**,** h** Migration ratio of BV2 cells towards fAβ (**f**) and oAβ (**g**). *n* = 3. NG: Normal glucose group. HG: High glucose group. TREM2-si: TREM2 knockdown group. HG + Vector: High-glucose group with empty vector. HG + TREM2-si: TREM2 knockdown high-glucose group. fAβ: Aβ fibril. oAβ: Aβ oligomer. Data were presented as mean ± SEM. For in vitro studies, n represents the number of biologically independent experiments performed. For c, two-tailed unpaired t test. For e, g, h, One-way ANOVA. * *p* < 0.05, ** *p* < 0.01, *** *p* < 0.001.

To investigate the effects of TREM2 on microglial migration under high glucose condition, we performed scratch and Transwell assays in vitro. In this study, TREM2 in BV2 cells was knocked down using siRNA, and the efficiency of the knockdown was confirmed by Western blot (*p* < 0.0001, Fig. 6b, c). At 48 h post-scratch, the migration ability of microglia in the high glucose (HG) group was significantly diminished compared to the normal glucose (NG) group (*p* = 0.0230, Fig. 6d, e). However, TREM2 knockdown did not alter the migration ability of microglia treated with high glucose (Fig. 6d, e). BrdU assays were used to evaluate the proliferation of BV2 cells. The results showed that neither high glucose nor TREM2 affected microglial proliferation (Fig. S5g).

In the Transwell assay, fAβ and oAβ were added separately to the lower chamber as chemoattractants. Compared to the NG group, the chemotactic response of microglia to both fAβ and oAβ was significantly reduced under high glucose condition (*p* < 0.0001, Fig. 6f-h). TREM2 knockdown did not affect the chemotactic response of high glucose-treated microglia to fAβ, but it significantly decreased the chemotactic response to oAβ (*p* = 0.0010, Fig. 6f-h). These findings indicate that TREM2 specifically affects the chemotaxis of microglia towards oAβ, but not fAβ, under high glucose condition.

### TREM2 promotes microglial phagocytosis of Aβ under high glucose condition

**Fig. 7:**
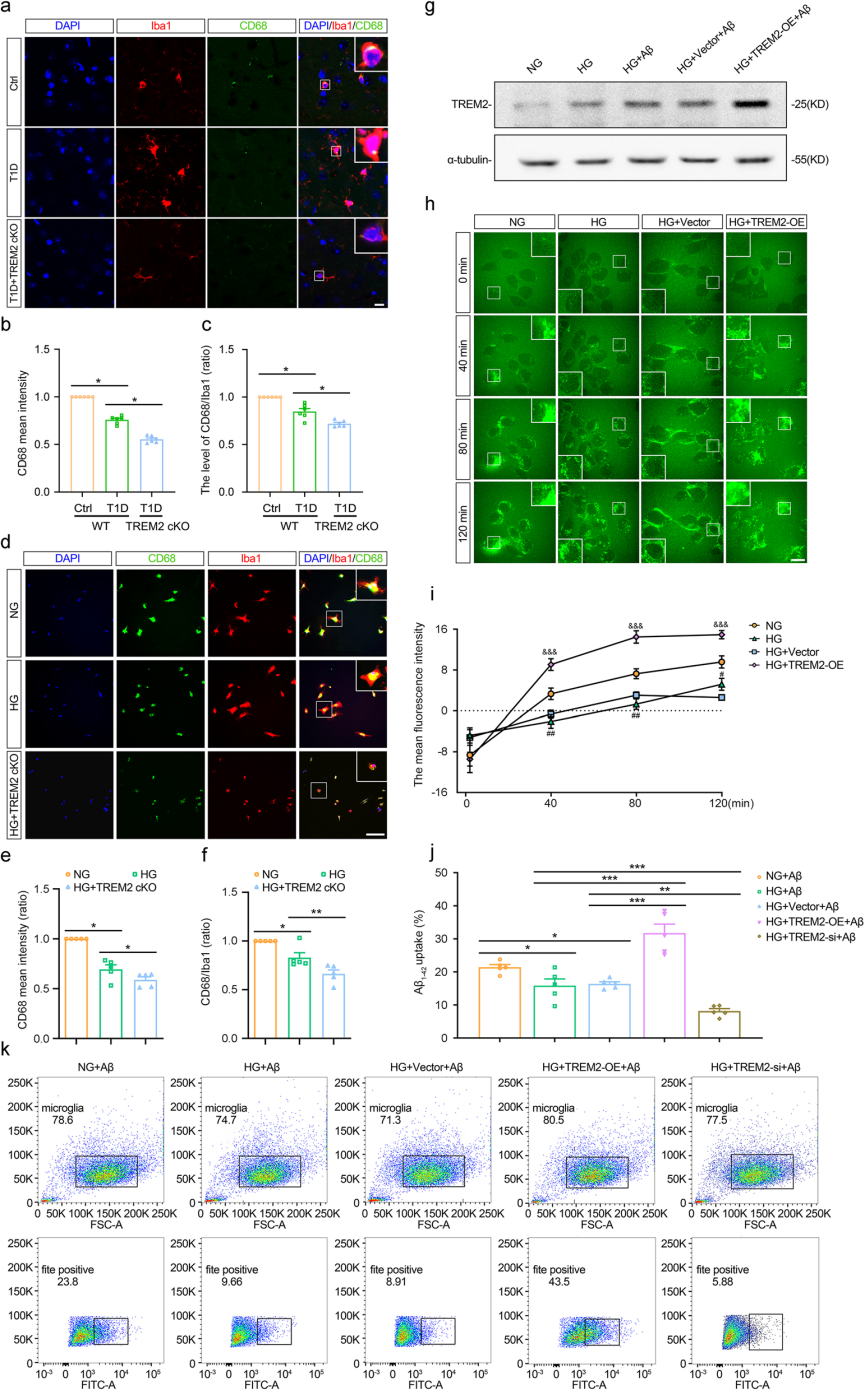
The impact of TREM2 on microglial phagocytosis of Aβ under T1D and high glucose condition. **a-c **Representative images of immunofluorescent staining (**a**) of Iba1 (red), CD68 (green) and DAPI (blue), CD68 mean intensity (**b**) and CD68/Iba-1 ratio (**c**) in the prefrontal cortex. Scale bar = 10 μm. n = 6. **d-f** Representative images of immunofluorescent staining (**d**) of Iba1 (red), CD68 (green) and DAPI (blue), CD68 mean intensity (**e**) and CD68/Iba-1 ratio (**f**) in primary cultured microglia. n = 5. **g** Representative images of Western blot showed the TREM2 expression in BV2 cells from different treatment groups. **h, i** Representative images (**h**) and mean intensity (**i**) of fluorescent Aβ (green) in BV2 cells at different time points. Scale bar = 7.8 μm. n = 3. **j, k** The percentage of fluorescent Aβ + BV2 cells (**j**) and representative flow cytometry dot plots (**k**). n = 5. WT: Wild-type. TREM2 cKO: TREM2 knockout. Ctrl: Wild-type nondiabetic group. T1D: Wild-type diabetic group. T1D + TREM2 cKO: TREM2 knockout diabetic group. NG: Normal glucose group. HG: High glucose group. HG + TREM2 cKO: TREM2 knockout high-glucose group. HG + Aβ: High-glucose group with Aβ. HG + Vector + Aβ: High-glucose group with empty vector and Aβ. HG + TREM2-OE + Aβ: High-glucose group with TREM2 overexpression and Aβ. HG + Vector: High-glucose group with empty-vector. HG + TREM2-OE: High-glucose group with TREM2 overexpression. NG + Aβ: Normal glucose group with Aβ. HG + TREM2-si + Aβ: High-glucose group with TREM2 knockdown and Aβ. Data were presented as mean ± SEM. For in vivo studies, n represents the number of animals per group. For in vitro studies, n represents the number of biologically independent experiments performed. For b, c, e, f, i, j, One-way ANOVA. * p < 0.05, ** p < 0.01, *** p < 0.001. # p < 0.05, ## p < 0.01, compared with NG + Aβ group. &&& p < 0.001, compared with HG + Vector + Aβ group.

In the prefrontal cortex, the intensity of CD68, a commonly used phagocytic marker, was significantly lower in wild-type T1D mice than in Ctrl mice, and was further reduced in T1D + TREM2 cKO mice (Fig. 7b: Ctrl vs. T1D: *p* < 0.0001, T1D vs. T1D + TREM2 cKO: *p* < 0.0001; Fig. 7c: Ctrl vs. T1D: *p* = 0.0002, T1D vs. T1D + TREM2 cKO: *p* = 0.0010, Fig. 7a-c). Similarly, TREM2 knockout further reduced CD68 intensity in primary cultured microglia under high glucose condition (Fig. 7e: NG vs. HG: *p* < 0.0001, HG vs. HG + TREM2 cKO: *p* = 0.0342; Fig. 7f: NG vs. HG: *p* = 0.0081, HG vs. HG + TREM2 cKO: *p* = 0.0088, Fig. 7d-f).

Live-cell imaging was employed to observe the dynamic process of microglia phagocytosing fluorescently labeled Aβ over a 2-hour period. Initially, no significant differences in Aβ fluorescence intensity in BV2 cells were observed among the groups. At 40, 80, and 120 min, high glucose treatment markedly decreased the fluorescence intensity of Aβ in microglia (40 min: *p* = 0.0009; 80 min: *p* = 0.0260, 120 min: *p* = 0.0198), whereas overexpression of TREM2 effectively reversed the high glucose-induced reduction in microglial phagocytosis of Aβ (40 min: *p* < 0.0001; 80 min: *p* < 0.0001, 120 min: *p* < 0.0001, Fig. 7h, i).

Parallel to these observations, flow cytometry results showed that under high glucose condition, TREM2 knockdown further reduced the phagocytosis of Aβ by BV2 cells (HG + Aβ vs. HG + TREM2-si + Aβ: *p* = 0.0243; HG + Vector + Aβ vs. HG + TREM2-si + Aβ: *p* = 0.0149, Fig. 7j, k). In contrast, TREM2 overexpression restored the phagocytic ability of BV2 cells diminished by high glucose (HG + Aβ vs. HG + TREM2-OE + Aβ: *p* < 0.0001; HG + Vector + Aβ vs. HG + TREM2-OE + Aβ: *p* = 0.0006, Fig. 7j, k). Similar trends were observed in N9 cells (Fig. S5f).

### Impact of TREM2 deficiency on gene expression in T1D mice

**Fig. 8:**
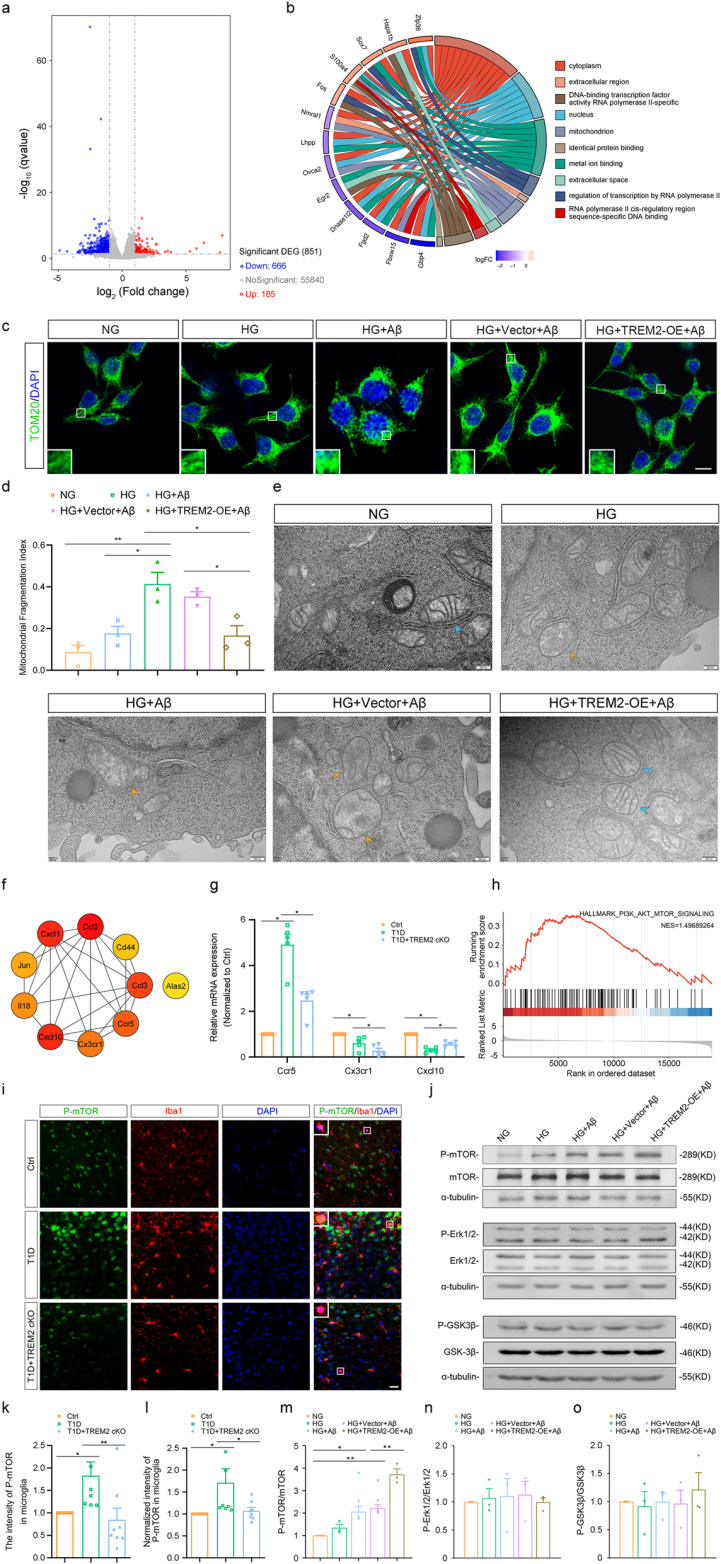
Identification and functional validation of differentially expressed genes related to TREM2 in the brain of T1D mice. **a** Volcano plot of differential gene expression profiles in the prefrontal cortex between T1D mice and T1D + TREM2 cKO mice. **b** Chord plot displaying the top 10 GO terms of differentially expressed genes. **c**,** d** Representative images of immunofluorescent staining (**c**) of TOM20 (green) and DAPI (blue), and the mitochondrial fragmentation index in BV2 cells, calculated as punctate/(punctate + rod-shaped + reticular) (**d**). Scale bar = 10 μm. *n* = 3. **e)** Representative electron microscopy images of mitochondria in BV2 cells. **f** Top 10 ranked hub gene networks generated by Cytohubba. **g** The relative mRNA levels of Ccr5, Cx3cr1, and Cxcl10 in the prefrontal cortex. *n* = 5. **h** GSEA showed significant enrichment in the PI3K/AKT/MTOR signaling pathway. **i**,** k**,** l** Representative images of immunofluorescent staining (**i**) of P-mTOR (green), Iba1 (red), and DAPI (blue), the intensity of P-mTOR in microglia (**k**), and normalized intensity of P-mTOR in microglia (**l**) in the prefrontal cortex. Scale bar = 10 μm. *n* = 8. **j**,** m-o** Representative images (**j**) and quantitative analysis of Western blot showed the levels of P-mTOR/mTOR (**m**), P-Erk1/2/Erk1/2 (**n**), and P-GSK3β/GSK3β (**o**) in BV2 cells. *n* = 3. Ctrl: Wild-type nondiabetic group. T1D: Wild-type diabetic group. T1D + TREM2 cKO: TREM2 knockout diabetic group. DEGs: Differentially expressed genes. GO: Gene ontology. GSEA: Gene set enrichment analysis. NG: Normal glucose group. HG: High glucose group. HG + Aβ: High-glucose group with Aβ. HG + Vector + Aβ: High-glucose group with empty vector and Aβ. HG + TREM2-OE + Aβ: High-glucose group with TREM2 overexpression and Aβ. Data were presented as mean ± SEM. For in vivo studies, n represents the number of animals per group. For in vitro studies, n represents the number of biologically independent experiments performed. For d, g, k-o, One-way ANOVA. * *p* < 0.05, ** *p* < 0.01

DEGs between wild-type T1D and T1D + TREM2 cKO mice were identified through bulk RNA-Seq. Volcano plots and heatmaps illustrated these differences, encompassing 185 upregulated and 666 downregulated genes (Fig. 8a, Fig. S6a). A PPI network revealed interactions among the DEGs, with the network consisting of 152 nodes and 158 edges (Fig. S6b). The top 10 hub genes, including Ccl2, Cxcl10, Cxcl1, Ccl3, Ccr5, Cx3cr1, Il18, Jun, Cd44 and Alas2, were identified using CytoHubba software (Fig. 8f). The qRT-PCR results confirmed that TREM2 deficiency increased the levels of Cxcl10 (T1D vs. T1D + TREM2 cKO: *p* = 0.0307, Fig. 8g) and decreased the levels of Ccr5 (T1D vs. T1D + TREM2 cKO: *p* = 0.0065, Fig. 8g) and Cx3cr1 (T1D vs. T1D + TREM2 cKO: *p* = 0.0030, Fig. 8g). GO analysis identified 144 GO terms significantly enriched with the DEGs, including 93 biological process (BP) terms, 22 cellular component (CC) terms and 29 molecular function (MF) terms. GO analysis indicated that these DEGs were primarily enriched in pathways related to innate immune response, lipid metabolism and apoptosis (BP category); in the cytoplasm, nucleus, extracellular region and mitochondria (CC category); and in functions associated with DNA binding, metal ion binding and cytokine activity (MF category) (Fig. S6c).The top 10 significantly enriched GO terms in the BP, CC and MF categories were shown in Fig. 8b and Supplementary Table 7, respectively. KEGG pathway analysis revealed that the DEGs were significantly enriched in metabolic and inflammation-related pathways, such as cytokine-cytokine receptor interaction, chemokine signaling, and TNF signaling (Fig. S6d). GSEA further disclosed significant enrichment of all genes in various biological and signaling pathways, including TNFA signaling via NF-κB, P53 pathway, hypoxia, inflammatory response, unfolded protein response and PI3K/AKT/mTOR signaling and so on (Fig. 8h, Fig. S6e, Supplementary Table 8). Heatmap illustrates the expression levels of DEGs involved in the PI3K/AKT/mTOR signaling pathway across samples (Fig. S6f).

### Protective effect of TREM2 on mitochondrial damage in microglia induced by high glucose and Aβ

Immunofluorescence analysis revealed that compared to the NG group, high glucose treatment resulted in an increasing trend in the mitochondrial fragmentation index (punctate/[reticular + rod-shaped + punctate]) of microglia, although the difference did not reach statistical significance (*p* = 0.1373; Fig. 8c, d). The combined high glucose and Aβ (HG + Aβ) treatment induced a more pronounced increase in mitochondrial fragmentation relative to the NG or HG treatments alone (NG vs. HG + Aβ: *p* = 0.0076, HG vs. HG + Aβ: *p* = 0.0200, Fig. 8c, d). However, overexpression of TREM2 in microglia markedly reduced the proportion of fragmented mitochondria induced by HG + Aβ co-treatment (HG + Aβ vs HG+ TREM2-OE +Aβ: *p* = 0.0017, HG + Aβ +Vector vs HG+TREM2-OE +Aβ: *p* = 0.0280, Fig. 8c, d).

Transmission electron microscopy (TEM) revealed that mitochondria in microglia of the NG group had normal morphology, appearing oval or spindle-shaped, with distinct inner and outer membranes and intact cristae. In contrast, the mitochondrial morphology in microglia treated with high glucose was altered, showing partial fragmentation or loss of cristae (as indicated by organe arrows in Fig. 8e). When exposed to both high glucose and Aβ, microglial mitochondria showed further degeneration, with swelling, deformation, cristae fragmentation, and flocculent or vacuolated areas (Fig. 8e, orange arrows). Overexpression of TREM2 improved mitochondrial morphology in microglia, reducing swelling, cristae fragmentation and vacuolated structures (Fig. 8e, blue arrows). These results suggest that high glucose and Aβ induce mitochondrial structural damage in microglia, and TREM2 can mitigate this damage.

To further investigate the functional consequences of mitochondrial damage, intracellular ROS levels were assessed using the fluorescent probe DCFH-DA. Compared with the NG group, microglia treated with high glucose showed significantly enhanced green fluorescence, indicating increased ROS production (NG vs. HG: *p* = 0.0002, Fig. S5h, i). Notably, TREM2 knockdown under high glucose conditions further augmented ROS generation, as evidenced by stronger fluorescence intensity (HG vs. HG + TREM2-si: *p* = 0.0358, Fig. S5h, i).

### TREM2 promotes mTOR activation in microglia under high glucose condition

To investigate the specific downstream signaling pathways by which TREM2 exerts its effects, we assessed the activation status of several key kinases, including mTOR, Erk1/2, and glycogen synthase kinase 3β (GSK3β), which are known to be implicated in TREM2 signaling and microglial function [53]. Western blot analysis in BV2 cells showed that compared to the NG group, mTOR phosphorylation levels exhibited an increasing trend after high glucose treatment alone (*p* = 0.0844), were significantly elevated by high glucose combined with Aβ treatment (NG vs. HG + Aβ: *p* = 0.0171), and were further enhanced by TREM2-OE (HG + Vector + Aβ vs. HG + TREM2-OE + Aβ: *p* = 0.0016; Fig. 8j, m). However, treatment with high glucose, Aβ and TREM2-OE did not affect the phosphorylation levels of Erk1/2 and GSK3β (Fig. 8j,n, o). These results suggest that TREM2 may modulate the function of microglia under high glucose condition via the mTOR signaling pathway, rather than through the Erk1/2 and GSK3β pathways.

Immunofluorescence results further substantiated these findings. The fluorescence intensity of p-mTOR in microglia was significantly higher in wild-type T1D mice than in Ctrl mice (Ctrl vs. T1D: *p* = 0.0142, Fig. 8i, k), but significantly lower in T1D + TREM2 cKO mice than in wild-type T1D mice (T1D vs. T1D + TREM2-cKO: *p* = 0.0255, Fig. 8i, k). To account for the increased number of microglia in the prefrontal cortex of wild-type T1D mice, we normalized the fluorescence intensity of p-mTOR to microglia count. The normalized analysis revealed that the fluorescence intensity of p-mTOR in individual microglia of wild-type T1D mice remained significantly greater than that in the Ctrl mice (Ctrl vs. T1D: *p* = 0.0477, Fig. 8i, l), whereas TREM2 knockout significantly reduced the fluorescence intensity (T1D vs. T1D + TREM2-cKO: *p* = 0.0175, Fig. 8i, l).

**Fig. 9:**
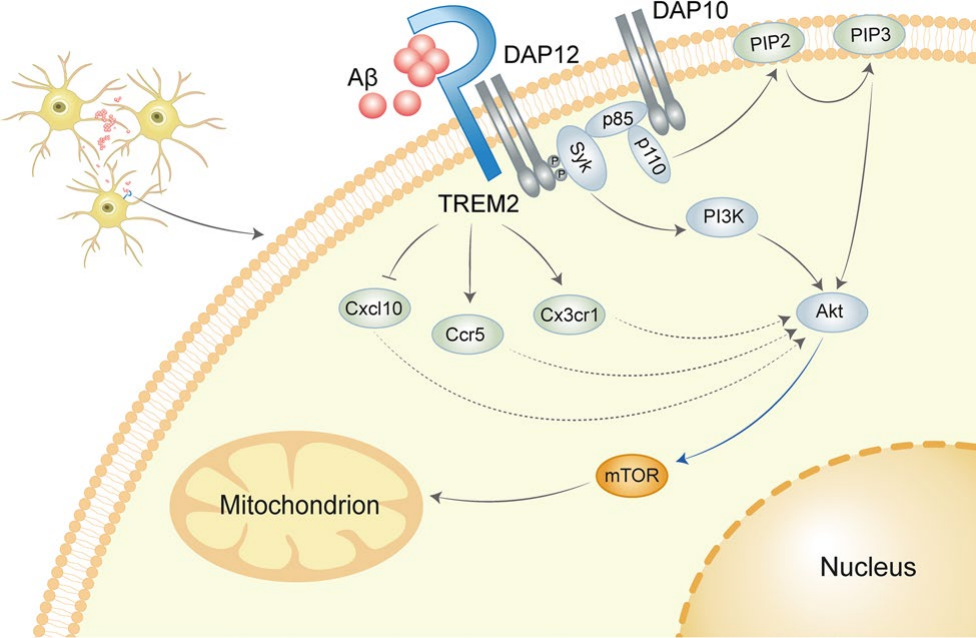
The TREM2-mTOR Axis Drives Microglial Clearance of Aβ in the Diabetic Brain. Accumulation of Aβ oligomers and upregulated TREM2 expression were observed in the prefrontal cortex of T1D mice. Under hyperglycemic conditions, Aβ activates TREM2, which phosphorylates its adaptor DAP12 and recruits Syk kinase. Syk then initiates the downstream PI3K-Akt-mTOR signaling pathway. This cascade enhances mitochondrial function and upregulates chemokines including Cxcl10, thereby enabling microglia to migrate toward, phagocytose, and clear Aβ oligomers efficiently. Collectively, these findings demonstrate that TREM2 plays a critical role in maintaining microglial homeostasis and countering Aβ pathology in T1D

Collectively, our findings support a model wherein TREM2 enhances microglial function through the mTOR signaling pathway, promoting migration, phagocytosis, and mitochondrial integrity, thereby facilitating Aβ clearance under hyperglycemic conditions (as summarized in Fig. 9).

## Discussion

This study investigated the cognitive consequences of T1D in murine models. Diabetic mice exhibited progressive learning and memory deficits across morris water and passive avoidance tests, with cognitive deterioration correlating with disease duration. Interestingly, CFC tests revealed comparable freezing times between diabetic and control groups, demonstrating preserved fear memory and indicating domain-specific rather than global cognitive impairment in T1D.

We further explored Aβ pathology. Although classical Aβ plaques were undetectable in T1D brains up to 25 weeks post-STZ induction, region-specific accumulation of neurotoxic Aβ oligomers was observed. In AD patients, the initial regions of Aβ deposition are typically the frontal lobes, medial temporal lobe (encompassing the hippocampus), and parietal, with a temporal progression to involve the diencephalon, striatum, brainstem, cerebellum, and other areas of the brain [54]. In contrast, in T1D mice, Aβ oligomer accumulation slightly differs. It first builded up in the prefrontal cortex, while the increase in the hippocampus wasn’t statistically significant. This spatial selectivity suggests the prefrontal cortex may serve as an early vulnerability site for Aβ oligomer accumulation in T1D, potentially preceding hippocampal involvement that may require extended disease progression or distinct pathological cascades. The observed Aβ dysregulation likely arises from synergistic interactions among disrupted insulin signaling, chronic hyperglycemia, and neuroinflammation [55–58]. which collectively promote Aβ precursor processing, oligomer stabilization, and impaired clearance mechanisms.

Notably, the low-molecular-weight oligomers (36–50 kDa) we observed differ from the predominant high-molecular-weight aggregates (56–400 kDa) reported in AD models [59] and human AD brain tissues [60]. This molecular dichotomy corresponds to distinct pathogenic mechanisms: Lower-weight oligomers preferentially disrupt intracellular homeostasis through membrane permeabilization, organelle dysfunction (e.g., endoplasmic reticulum stress, mitochondrial impairment), and aberrant signal transduction [61], whereas higher-weight oligomers predominantly drive extracellular plaque formation and synaptic network disruption [62]. This distinction implies potential mechanistic differences in Aβ aggregation processes between metabolic disorders like T1D and neurodegenerative conditions such as AD.

Emerging evidence indicates microglia exhibit dualistic responses to Aβ pathology—clearing soluble Aβ through phagocytic mechanisms while paradoxically amplifying neuroinflammation. In our T1D model, microglial proliferation and activation were first observed in the prefrontal cortex, coinciding with the accumulation of Aβ oligomers in this region. Additionally, T1D increased the colocalization of Aβ with microglia. These findings suggest that early microglial activation may be closely related to the Aβ pathology, particularly in the prefrontal cortex. Interestingly, this early activation phase (8 weeks post-STZ) coincided with suppressed phagocytic capacity, evidenced by reduced CD68 expression despite elevated Iba1 levels. Given that CD68 upregulation typically follows Iba1 during functional microglial maturation [63], our findings suggest T1D induces a maladaptive microglial state characterized by premature activation with impaired Aβ clearance. This dysfunction may perpetuate Aβ oligomer accumulation, creating a vicious cycle that exacerbates cognitive decline.

SnRNA-Seq techniques have significantly advanced the study of microglia, leading to the discovery of various novel subtypes. Our SnRNA-seq atlas of 59,356 cells from STZ-induced T1D mice revealed ten transcriptionally discrete microglial subpopulations response to metabolic stress. Our GO and KEGG analyses revealed distinct yet overlapping functional signatures among microglial subpopulations (M1, M2, M3, M5), highlighting both shared and specialized mechanisms in immune regulation, phagocytosis, and cellular metabolism. The Aβ-related genes (e.g., Trem2, CCR5, and Cx3cr1) are predominantly found in the M1, M2, M3 amd M5 subclusters. The expression levels of Trem2, CCR5, and Cx3cr1 are significantly elevated in the T1D group. Pseudotime trajectory analysis indicates a gradual downregulation of Trem2, CCR5, and Cx3cr1, suggesting that chronic hyperglycemia induces functional exhaustion of microglia. This transcriptional attenuation is parallel to the timeline of Aβ oligomer accumulation observed in T1D mice, proposing a failed compensatory mechanism, in which the initial activation of microglial phagocytosis is ultimately disrupted by diabetic metabolic disturbances.

TREM2, a microglia-specific receptor in the CNS [17], was significantly upregulated in T1D mouse brains, particularly in M1. Notably, this upregulation exhibited spatiotemporal specificity: TREM2 expression increased earlier in the prefrontal cortex than in the hippocampus, paralleling the regional accumulation patterns of Aβ oligomers in T1D mice. This spatiotemporal correlation raises the possibility that TREM2 induction may be triggered by Aβ pathology, consistent with studies showing TREM2’s role in binding and promoting Aβ clearance [17, 64]. Importantly, TREM2 knockout exacerbated cognitive deficits and prefrontal Aβ burden in T1D mice, mirroring observations in AD models where TREM2 activation enhances microglial plaque encapsulation, Aβ phagocytosis, and cognitive recovery [65, 66]. Together, these findings suggest that TREM2 upregulation in T1D brains represents a compensatory response to counteract dysfunctional microglial activity. The migratory and phagocytic capacities of microglia constitute essential mechanisms for Aβ clearance. Our investigation reveals context-dependent effects of TREM2 on microglial trafficking in T1D models. In vivo analyses demonstrated impaired migration of TREM2-deficient microglia toward 6E10-positive neurons within the prefrontal cortex of T1D mice. Surprisingly, this phenotype was not recapitulated in scratch assays under hyperglycemic conditions, suggesting microenvironment-specific regulation of TREM2-mediated motility. Further transwell chemotaxis assays delineated differential Aβ-form selectivity: TREM2 knockdown specifically attenuated microglial migration toward Aβ oligomers, but not Aβ fibrils, under hyperglycemic stress. The preserved migration in scratch assays (measuring undirected motility) versus impaired chemotaxis in transwell systems (assessing directed migration) suggests TREM2 predominantly regulates signal-guided trafficking rather than basal motility. The retained response to Aβ fibrils implies distinct receptor machinery governs microglial recognition of fibrillar versus oligomeric Aβ species, with TREM2 playing a predominant role in oligomer-directed chemotaxis under diabetic conditions.

TREM2 is a well-established regulator of microglial phagocytosis in neurodegenerative contexts. Prior studies demonstrate that TREM2 knockout reduces CD68 (a lysosomal phagocytic marker) expression in aged or demyelinated mouse brains [67, 68], and impairs Aβ plaque clearance via dampened microglial engulfment [69, 70], whereas TREM2 activation enhances Aβ phagocytosis in AD models [65, 66]. Our findings extend these observations to T1D-associated neurodegeneration: TREM2 knockout exacerbated the T1D-induced CD68 downregulation in microglia in the prefrontal cortex, suggesting a conserved role of TREM2 in sustaining phagocytic capacity under metabolic stress. However, existing studies predominantly focus on fibrillar Aβ plaques, overlooking the neurotoxic Aβ oligomers that dominate early AD/T1D pathology. Using flow cytometry and live-cell imaging, we uncovered a novel oligomer-selective phagocytic role of TREM2: TREM2 significantly enhanced microglial uptake of Aβ oligomers, but this protective mechanism was blunted under high glucose conditions.

Integrative transcriptomic profiling through bulk RNA-Seq revealed profound dysregulation of neuroinflammatory and metabolic pathways mediated by TREM2 deficiency in the T1D brain. GO analysis revealed that DEGs were enriched in immune response and inflammation-related pathways, which is consistent with the chronic inflammatory state observed in diabetic encephalopathy [71]. KEGG pathway mapping further delineated hyperactivation of cytokine-cytokine receptor interactions, chemokine signaling, and TNF-mediated inflammatory cascades. These findings collectively suggest that TREM2 loss sustains microglial pro-inflammatory polarization via constitutive activation of innate immune signaling, thereby amplifying neuroinflammatory tissue injury and accelerating neurodegenerative cascades in diabetic contexts. Notably, our pathway analysis uncovered novel intersections between TREM2 deficiency and metabolic dysregulation, with DEGs prominently enriched in lipid catabolism and apoptotic signaling pathways.

GO functional enrichment analysis demonstrates significant associations between TREM2 knockout and mitochondrial dysregulation in microglia. Microglia depend on mitochondrial bioenergetics to execute essential neuroprotective functions, including Aβ plaque clearance and synaptic pruning through phagocytic activity [72]. Emerging evidence indicates that mitochondrial dynamics (fusion/fission balance) directly regulate microglial phenotypes and subsequent neuroinflammatory responses [73], establishing mitochondrial integrity as a critical determinant of glial pathophysiology. While previous studies have documented hyperglycemia-induced mitochondrial impairment in pericytes [74] and TREM2-related mitochondrial deficits in Schwann cells [75], the microglial mitochondrial dysfunction under hyperglycemic conditions remain underexplored. Our experimental data demonstrate that high glucose and Aβ exposure induced severe mitochondrial damage in microglia, which was effectively mitigated by TREM2 overexpression. Moreover, TREM2 knockdown exacerbated oxidative stress under high glucose conditions. Collectively, these findings highlight the protective role of TREM2 in preserving mitochondrial structure and reducing oxidative damage, underscoring its importance in maintaining microglial bioenergetic homeostasis under diabetic and Aβ-stressed conditions.

Our GSEA analysis of T1D murine brain transcriptomes revealed significant enrichment of PI3K/Akt/mTOR pathway components following TREM2 ablation, a finding corroborated by biochemical assays demonstrating TREM2-dependent mTOR activation under sustained hyperglycemia without concurrent modulation of Erk1/2 or GSK3β phosphorylation states. The identified TREM2-mTOR axis exhibits critical functional implications: Pharmacological mTOR activation enhances microglial Aβ clearance efficiency and reduces synaptic density loss in 5XFAD models [76], while conditional mTOR knockout impairs neuronal debris phagocytosis [77]. In AD patients with TREM2 risk variants and in TREM2-deficient AD mice, defective mTOR signaling leads to an abundance of autophagic vesicles, thereby exacerbating Aβ pathology. Furthermore, mTOR also promotes mitochondrial biogenesis by phosphorylating various substrates, affecting mitochondrial metabolism and dynamics. Future studies should further explore the role of the TREM2-mTOR axis in regulation of microglial function, as well as how to target this axis to improve the pathological conditions associated with T1D.

This study has several limitations. First, the experiments were conducted exclusively in male mouse models. This design was based on the consideration that cyclical variations in sex hormones may directly regulate microglial function and influence neuroinflammatory cascades [78, 79]. However, this approach may limit the generalizability of our findings to female T1D patients. Future studies are warranted to validate the regulatory role of TREM2 and its functional outcomes in female models, which is crucial for a comprehensive understanding of its pathophysiology and therapeutic relevance. Second, to model uncontrolled diabetic conditions and mechanistically elucidate the direct impact of chronic hyperglycemia on CNS pathologies, insulin intervention was not implemented in our STZ-induced T1D mice. While this approach avoids the confounding effects of exogenous insulin and effectively isolates hyperglycemia-induced mechanisms, it does not fully recapitulate the clinical scenario where the majority of T1D patients are under insulin therapy. Consequently, the therapeutic efficacy of TREM2 modulation needs to be further validated in insulin-treated models to better assess its clinical relevance. Finally, although our results indicate the involvement of the mTOR signaling pathway in TREM2-mediated cognitive improvement, this mechanism has not yet been validated in more human disease-relevant in vitro models, such as induced pluripotent stem cell (iPSC)-derived microglia. This limitation should be addressed in subsequent research.

## Conclusion

In this study, we conducted snRNA-seq on 59,356 cells from the prefrontal cortex and hippocampus of STZ-induced T1D and Ctrl mice. We identified ten microglial subpopulations, with Trem2-enriched clusters showing impaired phagocytosis and metabolic dysregulation. T1D mice exhibited progressive memory deficits and prefrontal Aβ oligomer accumulation, alongside region-specific microglial activation. TREM2 knockout exacerbated cognitive decline and Aβ pathology. Mechanistically, TREM2 regulated microglial migration, Aβ phagocytosis, and mitochondrial integrity under high-glucose conditions via mTOR signaling. These findings establish TREM2 as a critical regulator of microglial Aβ clearance in T1D, orchestrating mitochondrial and phagocytic functions through mTOR. This highlights TREM2’s therapeutic potential for mitigating diabetic neurodegeneration by enhancing microglial function and Aβ clearance.

## Supplementary Information

Supplementary Material 1.

Supplementary Material 2.

Supplementary Material 3.

Supplementary Material 4.

Supplementary Material 5.

Supplementary Material 6.

Supplementary Material 7.

Supplementary Material 8.

Supplementary Material 9.

## Data Availability

All relevant data associated with the published study are present in the paper or the Supplementary Information. The source data underlying the main and Supplementary Figs. are provided as a Source Data file. Our snRNA-seq and RNA-seq have been uploaded to the China National Center for Bioinformation (CNCB) and can be found under the accession number CRA031520.
