# Supplementary material for: Cognitive dysfunction in type 1 diabetes: role of TREM2 in microglial activation and Aβ pathology
Source: J Neuroinflammation. 2026 Jan 2;23:15. doi: 10.1186/s12974-025-03611-3 (PMC12801531; doi:10.1186/s12974-025-03611-3)
Supplement: Supplementary file 1 — Supplementary Material 1. [file 12974_2025_3611_MOESM1_ESM.docx]

**Table 1. Drugs and reagents used in this study**

| **Name** | **Company** | **Country** |
| --- | --- | --- |
| Streptozotocin | Sigma-Aldrich | USA |
| Aβ oligomers (oAβ) | GL Biochem | China |
| Aβ fibrils (fAβ) | ChinaPeptides | China |
| DMEM/F12 | Gibco | USA |
| Fetal Bovine Serum (FBS) | Gibco | USA |
| Penicillin-Streptomycin | KeyGEN BioTECH | China |
| Trypsin (0.25%) | KeyGEN BioTECH | China |
| Fluorescently labeled Aβ | GL Biochem | China |
| ROS Assay Kit | Beyotime | China |
| Mouse Aβ1-42 ELISA Kit | AnaSpec | China |
