# Supplementary material for: Cognitive dysfunction in type 1 diabetes: role of TREM2 in microglial activation and Aβ pathology
Source: J Neuroinflammation. 2026 Jan 2;23:15. doi: 10.1186/s12974-025-03611-3 (PMC12801531; doi:10.1186/s12974-025-03611-3)
Supplement: Supplementary file 2 — Supplementary Material 2. [file 12974_2025_3611_MOESM2_ESM.docx]

**Table 2. Instruments used in this study**

| **Name** | **Company** | **Country** |
| --- | --- | --- |
| Flow Cytometer | Miltenyi Biotec | Germany |
| Chromium Single Cell 3' Chip | 10x Genomics | USA |
| Illumina NovaSeq 6000 | Illumina | USA |
| Laser Scanning Confocal Microscope | Leica | Germany |
| Fusion FX6 XT Imaging System | Vilber Lourmat | France |
| QuantStudio 5 Real-Time PCR System | ABI | USA |
| MyCycler PCR Amplifier | Bio-rad | USA |
