# Supplementary material for: Cognitive dysfunction in type 1 diabetes: role of TREM2 in microglial activation and Aβ pathology
Source: J Neuroinflammation. 2026 Jan 2;23:15. doi: 10.1186/s12974-025-03611-3 (PMC12801531; doi:10.1186/s12974-025-03611-3)
Supplement: Supplementary file 3 — Supplementary Material 3. [file 12974_2025_3611_MOESM3_ESM.docx]

**Table 3. Antibodies used in this study**

| **Name** | **Source** | **Dilution** | **Company** | **Country** |
| --- | --- | --- | --- | --- |
| anti-Aβ1-42 | rabbit | 1:500 | abcam | USA |
| anti-Iba1 | rabbit | 1:500 | abcam | USA |
| anti-TREM2 | goat | 1:500 | abcam | USA |
| anti-GAPDH | mosue | 1:500 | Sigma | USA |
| anti-β-actin | rabbit | 1:500 | ABclonal | China |
| anti-mTOR | rabbit | 1:500 | Sigma | USA |
| anti-phospho-mTOR | rabbit | 1:500 | Sigma | USA |
| anti-ERK1/2 | rabbit | 1:500 | abcam | USA |
| anti-phospho-ERK1/2 | rabbit | 1:500 | abcam | USA |
| anti-GSK3β | mouse | 1:500 | abcam | USA |
| anti-phospho-GSK3β | rabbit | 1:500 | Sigma | USA |
| anti-α-tubulin | mouse | 1:500 | abcam | USA |
| anti-6E10 | mouse | 1:500 | Biolegend | USA |
| anti-CD68 | rat | 1:500 | Bio-rad | USA |
| anti-MAP2 | mouse | 1:500 | abcam | USA |
| anti-Rabbit IgG | goat | 1:4000 | Thermo Fisher Scientific | USA |
| anti-Mouse IgG | goat | 1:4000 | Jackson ImmunoResearch | USA |
| anti-Goat IgG | donkey | 1:4000 | Jackson ImmunoResearch | USA |
