# Supplementary material for: Cognitive dysfunction in type 1 diabetes: role of TREM2 in microglial activation and Aβ pathology
Source: J Neuroinflammation. 2026 Jan 2;23:15. doi: 10.1186/s12974-025-03611-3 (PMC12801531; doi:10.1186/s12974-025-03611-3)
Supplement: Supplementary file 4 — Supplementary Material 4. [file 12974_2025_3611_MOESM4_ESM.docx]

**Table 4. Cell seeding densities used in this study**

| **Experiment Type** | **Cell density (cells/mL)** |
| --- | --- |
| Western Blot/qPCR | 2×10^6^ |
| lmmunofluorescence | 1×10^5^ |
| Wound scratch | 1×10^5^ |
| Transwell assay | 1×10^5^ |
| Flow cytometry | 1×10^6^ |
| living cell imaging | 1×10^5^ |
| Brdu assay | 2×10^5^ |
