# Supplementary material for: Cognitive dysfunction in type 1 diabetes: role of TREM2 in microglial activation and Aβ pathology
Source: J Neuroinflammation. 2026 Jan 2;23:15. doi: 10.1186/s12974-025-03611-3 (PMC12801531; doi:10.1186/s12974-025-03611-3)
Supplement: Supplementary file 5 — Supplementary Material 5. [file 12974_2025_3611_MOESM5_ESM.docx]

**Table 5. Image acquisition parameters for immunofluorescence using laser scanning confocal microscopy.**

| Channel | Laser Wavelength (nm) | Laser Power (%) | Exposure Time (ms) |
| --- | --- | --- | --- |
| Blue | 405 | 0.5 | 100 |
| Green | 488 | 3 | 100 |
| Red | 594 | 3 | 100 |
