# Supplementary material for: Cognitive dysfunction in type 1 diabetes: role of TREM2 in microglial activation and Aβ pathology
Source: J Neuroinflammation. 2026 Jan 2;23:15. doi: 10.1186/s12974-025-03611-3 (PMC12801531; doi:10.1186/s12974-025-03611-3)
Supplement: Supplementary file 9 — Supplementary Material 9. [file 12974_2025_3611_MOESM9_ESM.pdf]

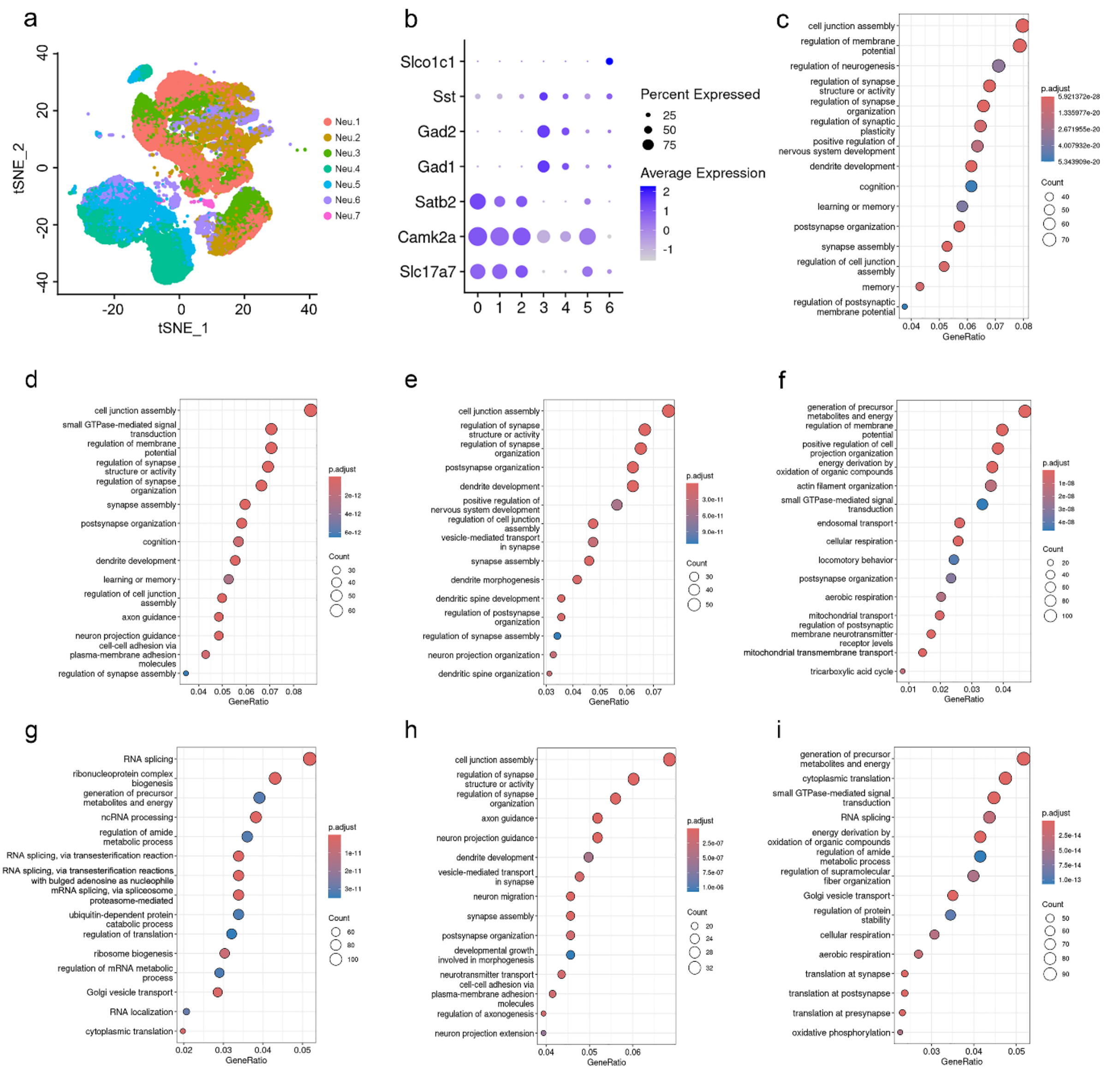

sFig. 1 Characterization and functional analysis of neuron subpopulations in T1D mice

a) tSNE plot showing the neuron subpopulations. b) Bubble plot showing the expression of the established marker genes for each neuron subpopulation. c-i) Top 15 GO terms enrichment in each neuron subpopulation. Neu: Neuron. Ctrl: Wild-type nondiabetic group. T1D: Wild-type diabetic group, GO: Gene ontology.

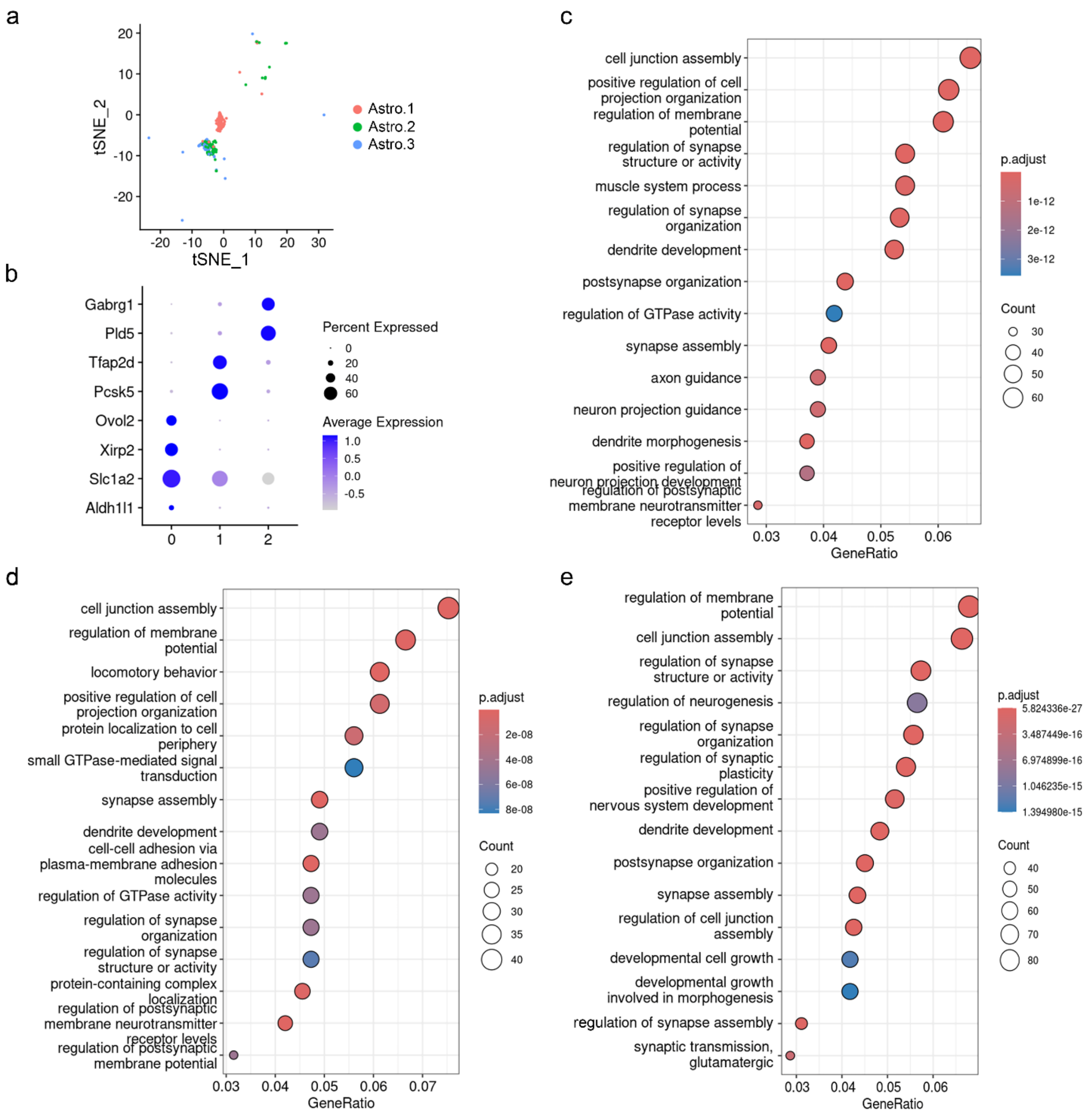

sFig. 2 Characterization and functional analysis of astrocyte subpopulations in T1D mice

a) tSNE plot showing the astrocyte subpopulations. b) Bubble plot showing the expression of the established marker genes for each astrocyte subpopulation. c-e) Top 15 GO terms enrichment in each astrocyte subpopulation. Astro: Astrocyte. Ctrl: Wild-type nondiabetic group. T1D: Wild-type diabetic group, GO: Gene ontology.

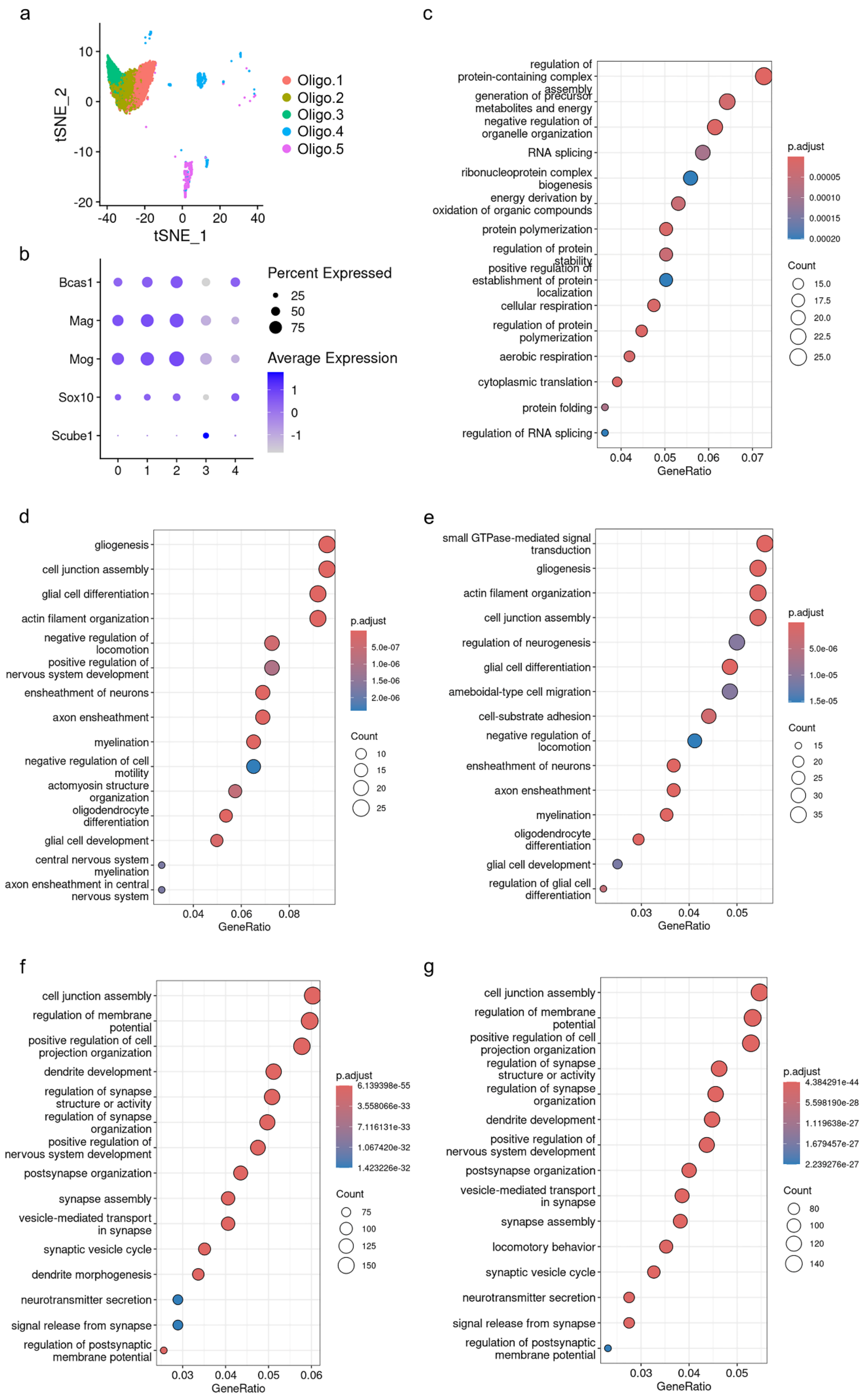
a
tSNE_2
tSNE_1
10
0
-10
-20
-40
-20
0
20
40
Oligo.1
Oligo.2
Oligo.3
Oligo.4
Oligo.5
b
Bcas1
Mag
Mog
Sox10
Scube1
0
1
2
3
4
Percent Expressed
25
50
75
Average Expression
1
0
-1
c
regulation of protein-containing complex assembly
generation of precursor metabolites and energy
negative regulation of organelle organization
RNA splicing
ribonucleoprotein complex biogenesis
energy derivation by oxidation of organic compounds
protein polymerization
regulation of protein stability
positive regulation of establishment of protein localization
cellular respiration
regulation of protein polymerization
aerobic respiration
cytoplasmic translation
protein folding
regulation of RNA splicing
0.04
0.05
0.06
0.07
GeneRatio
p.adjust
0.00005
0.00010
0.00015
0.00020
Count
15.0
17.5
20.0
22.5
25.0
d
gliogenesis
cell junction assembly
glial cell differentiation
actin filament organization
negative regulation of locomotion
positive regulation of nervous system development
ensheathment of neurons
axon ensheathment
myelination
negative regulation of cell motility
actomyosin structure organization
oligodendrocyte differentiation
glial cell development
central nervous system myelination
axon ensheathment in central nervous system
0.04
0.06
0.08
GeneRatio
p.adjust
5.0e-07
1.0e-06
1.5e-06
2.0e-06
Count
10
15
20
25
e
small GTPase-mediated signal transduction
gliogenesis
actin filament organization
cell junction assembly
regulation of neurogenesis
glial cell differentiation
ameboidal-type cell migration
cell-substrate adhesion
negative regulation of locomotion
ensheathment of neurons
axon ensheathment
myelination
oligodendrocyte differentiation
glial cell development
regulation of glial cell differentiation
0.03
0.04
0.05
GeneRatio
p.adjust
5.0e-06
1.0e-05
1.5e-05
Count
15
20
25
30
35
f
cell junction assembly
regulation of membrane potential
positive regulation of cell projection organization
dendrite development
regulation of synapse structure or activity
regulation of synapse organization
positive regulation of nervous system development
postsynapse organization
synapse assembly
vesicle-mediated transport in synapse
synaptic vesicle cycle
dendrite morphogenesis
neurotransmitter secretion
signal release from synapse
regulation of postsynaptic membrane potential
0.03
0.04
0.05
0.06
GeneRatio
p.adjust
6.139398e-55
3.558066e-33
7.116131e-33
1.067420e-32
1.423226e-32
Count
75
100
125
150
g
cell junction assembly
regulation of membrane potential
positive regulation of cell projection organization
regulation of synapse structure or activity
regulation of synapse organization
dendrite development
positive regulation of nervous system development
postsynapse organization
vesicle-mediated transport in synapse
synapse assembly
locomotory behavior
synaptic vesicle cycle
neurotransmitter secretion
signal release from synapse
regulation of postsynaptic membrane potential
0.03
0.04
0.05
GeneRatio
p.adjust
4.384291e-44
5.598190e-28
1.119638e-27
1.679457e-27
2.239276e-27
Count
80
100
120
140

sFig. 3 Characterization and functional analysis of oligodendrocyte subpopulations in T1D mice

a) tSNE plot showing the oligodendrocyte subpopulations. b) Bubble plot showing the expression of the established marker genes for each oligodendrocyte subpopulation. c-g) Top 15 GO terms enrichment in each oligodendrocyte subpopulation. Oligo: Oligodendrocyte. Ctrl: Wild-type nondiabetic group. T1D: Wild-type diabetic group, GO: Gene ontology.

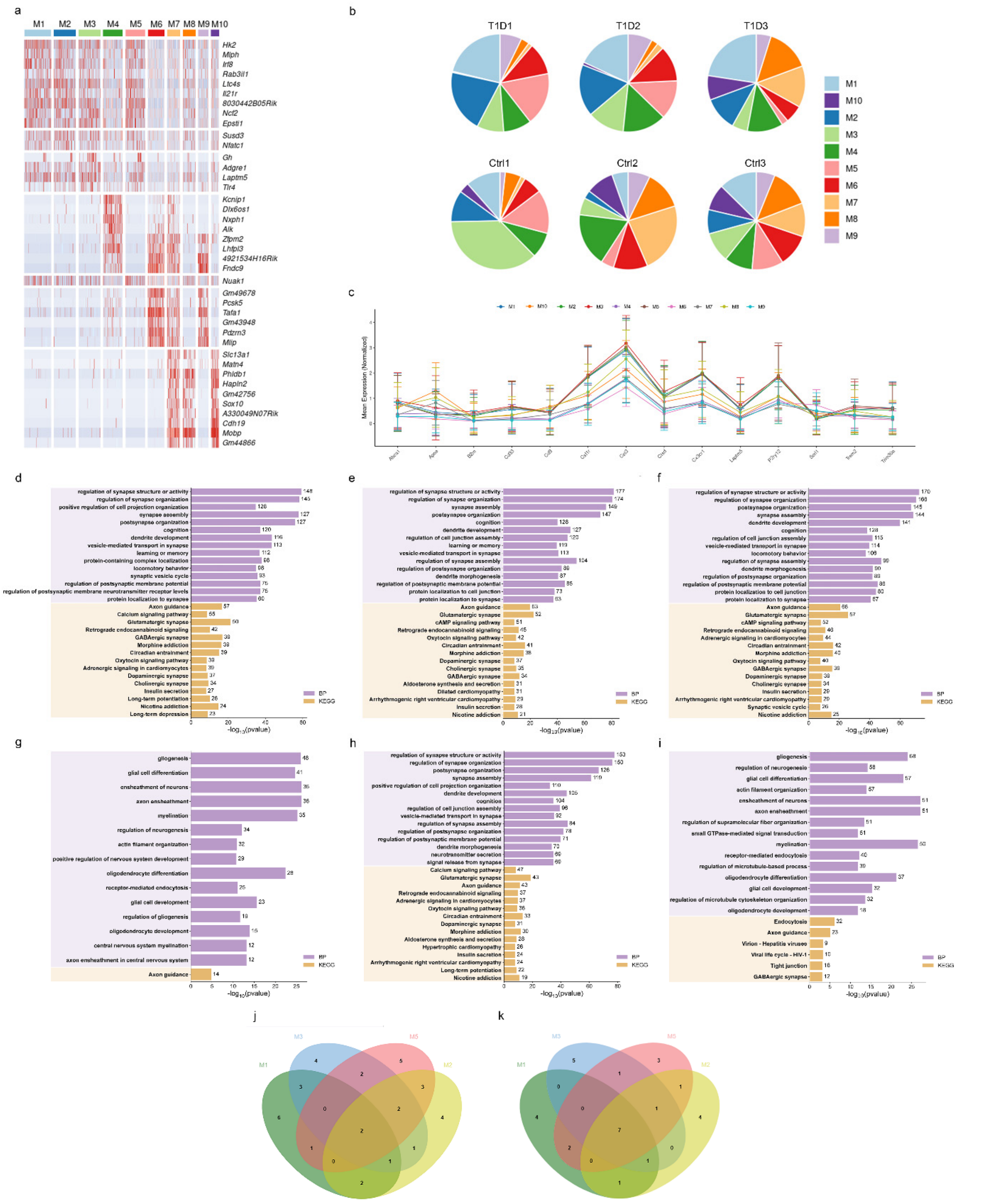

sFig. 4 Comparative analysis of microglial subpopulations in T1D mice.

a) Heatmap of established marker genes for each microglia subpopulation. **b)** Pie plot showing the relative proportion of microglia subpopulations in each sample. **c)** Line graph of the expression of established marker genes across each microglia subpopulation. **d-i)** Top 15

significantly enriched GO terms in biological process and top 15 significantly enriched KEGG pathways in differentially expressed genes for subpopulations of M4 (d), M6 (e), M7 (f), M8 (g), M9 (h), and M10 (i). **j-k)** Venn diagrams representing the number of shared and unique GO terms (j) and KEGG pathways (k) among subpopulations of M1, M2, M3, and M5. M: Microglia, Ctrl: Wild-type nondiabetic group, T1D: Wild-type diabetic group, GO: Gene ontology, KEGG: Kyoto encyclopedia of genes and genomes.

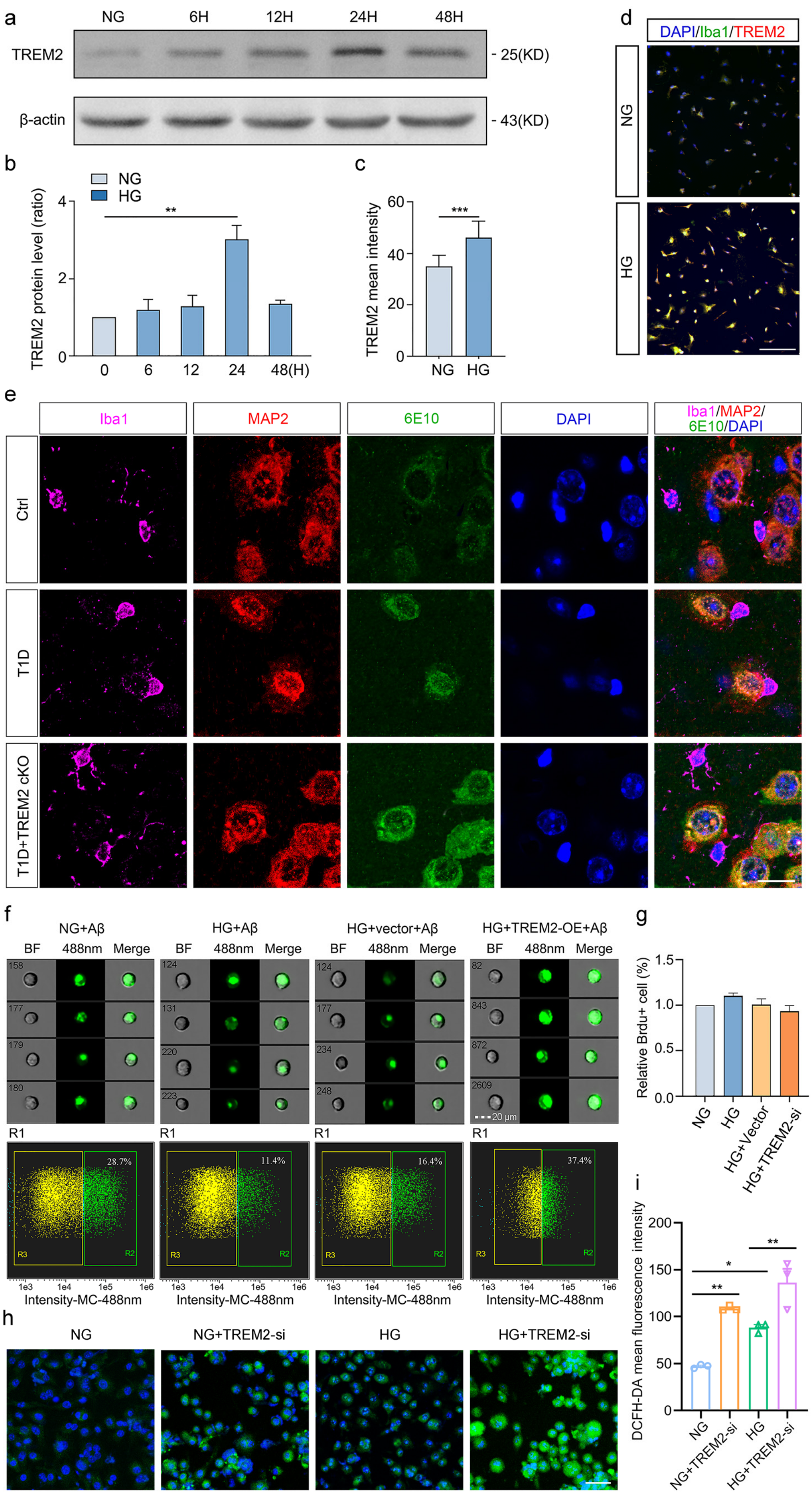

sFig. 5 TREM2 expression and functional impact in high glucose-treated microglia.

a, b) Representative images (a) and quantitative analysis (b) of Western blot showed the level of TREM2 in BV2 cells under high glucose treatment for different time points. n = 3. **c, d)** Mean intensity of TREM2 (c) and representative images of immunofluorescent staining (d) of Iba1 (green), TREM2 (red) and DAPI (blue) in BV2 cells. Scale bar = 100 µm. n = 3. **e)** Representative images of immunofluorescent staining of Iba1 (purple), MAP2 (red), 6E10 (green) and DAPI (blue) in BV2 cells. Scale bar = 10 µm. **f)** Representative flow cytometry dot plots of fluorescent $A\beta^{+}$ N9 cells. **g)** Percentage of BrdU+ cells of each group. n = 3. (h) Representative images and (i) quantification of DCFH-DA mean fluorescence intensity. DCFH-DA (green, ROS) and DAPI (blue, nuclei). Scale bar = 50 µm. n = 3. NG: Normal glucose group. HG: High glucose group. Ctrl: Wild-type nondiabetic group. T1D: Wild-type diabetic group. T1D + TREM2 cKO: TREM2 knockout diabetic group. NG+ Aβ: Normal glucose group with Aβ. HG+ Aβ: High-glucose group with Aβ. HG + Vector + Aβ: High-glucose group with empty vector and Aβ. HG+TREM2-OE+Aβ: High-glucose group with TREM2 overexpression and Aβ. NG+TREM2-si: TREM2 knockdown normal-glucose group. HG+TREM2-si: TREM2 knockdown high-glucose group. Data were presented as mean ± SEM. For in vitro studies, n represents the number of biologically independent experiments performed. For c, unpaired t-test. For b, g, i, one-way ANOVA. * $p<0.05$, ** $p<0.01$, *** $p<0.001$.

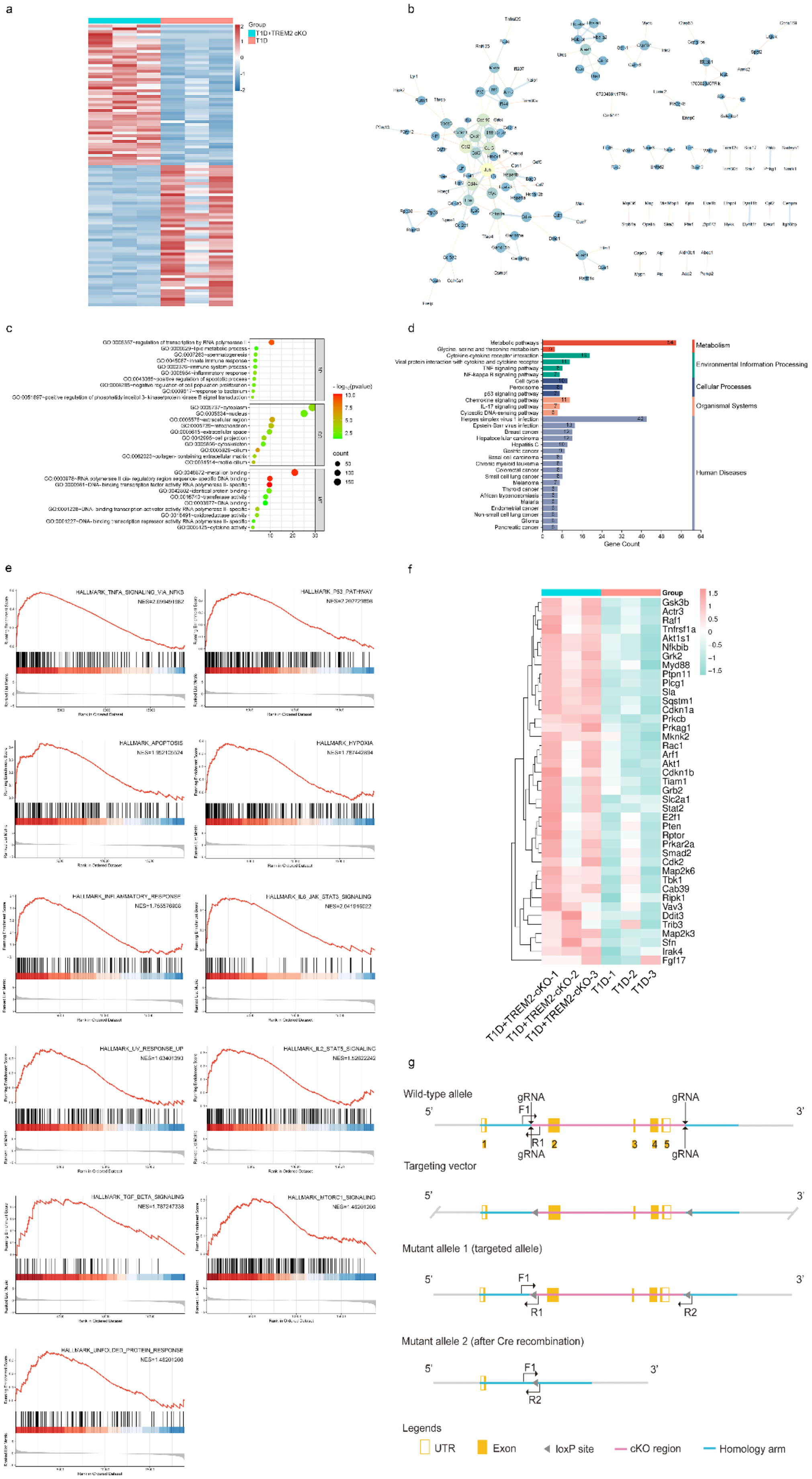

a
Group
T1D+TREM2 cKO
T1D
b
c
GO:0006357~regulation of transcription by RNA polymerase II
GO:0006629~lipid metabolic process
GO:0007283~spermatogenesis
GO:0045087~innate immune response
GO:0002376~immune system process
GO:0006954~inflammatory response
GO:0043065~positive regulation of apoptotic process
GO:0008285~negative regulation of cell population proliferation
GO:0009617~response to bacterium
GO:0051897~positive regulation of phosphatidylinositol 3-kinase/protein kinase B signal transduction
GO:0005737~cytoplasm
GO:0005634~nucleus
GO:0005576~extracellular region
GO:0005739~mitochondrion
GO:0005615~extracellular space
GO:0042995~cell projection
GO:0005856~cytoskeleton
GO:0005929~cilium
GO:0062023~collagen-containing extracellular matrix
GO:0031514~motile cilium
GO:0046872~metal ion binding
GO:0000978~RNA polymerase II cis-regulatory region sequence-specific DNA binding
GO:0000981~DNA-binding transcription factor activity RNA polymerase II-specific
GO:0042802~identical protein binding
GO:0016740~transferase activity
GO:0003677~DNA binding
GO:0001228~DNA-binding transcription activator activity RNA polymerase II-specific
GO:0016491~oxidoreductase activity
GO:0001227~DNA-binding transcription repressor activity RNA polymerase II-specific
GO:0005125~cytokine activity
BP
CC
MF
-log10(pvalue)
count
d
Metabolic pathways
Glycine, serine and threonine metabolism
Cytokine-cytokine receptor interaction
Viral protein interaction with cytokine and cytokine receptor
TNF signaling pathway
NF-kappa B signaling pathway
Cell cycle
Peroxisome
p53 signaling pathway
Chemokine signaling pathway
IL-17 signaling pathway
Cytosolic DNA-sensing pathway
Herpes simplex virus 1 infection
Epstein-Barr virus infection
Breast cancer
Hepatocellular carcinoma
Hepatitis C
Gastric cancer
Basal cell carcinoma
Chronic myeloid leukemia
Colorectal cancer
Small cell lung cancer
Melanoma
Thyroid cancer
African trypanosomiasis
Malaria
Endometrial cancer
Non-small cell lung cancer
Glioma
Pancreatic cancer
Metabolism
Environmental Information Processing
Cellular Processes
Organismal Systems
Human Diseases
Gene Count
e
HALLMARK_TNFA_SIGNALING_VIA_NFKB
HALLMARK_P53_PATHWAY
HALLMARK_APOPTOSIS
HALLMARK_HYPOXIA
HALLMARK_INFLAMMATORY_RESPONSE
HALLMARK_IL6_JAK_STAT3_SIGNALING
HALLMARK_UV_RESPONSE_UP
HALLMARK_IL2_STAT5_SIGNALING
HALLMARK_TGF_BETA_SIGNALING
HALLMARK_MTORC1_SIGNALING
HALLMARK_UNFOLDED_PROTEIN_RESPONSE
f
Group
Gsk3b
Actr3
Raf1
Tnfrsf1a
Akt1s1
Nfkbib
Grk2
Myd88
Ptpn11
Plcg1
Sla
Sqstm1
Cdkn1a
Prkcb
Prkag1
Mknk2
Rac1
Arf1
Akt1
Cdkn1b
Tiam1
Grb2
Slc2a1
E2f1
Stat2
Pten
Rptor
Prkar2a
Smad2
Cdk2
Map2k6
Tbk1
Cab39
Ripk1
Vav3
Ddit3
Trib3
Map2k3
Sfn
Irak4
Fgf17
T1D+TREM2-cKO-1
T1D+TREM2-cKO-2
T1D+TREM2-cKO-3
T1D-1
T1D-2
T1D-3
g
Wild-type allele
gRNA
F1
R1
1
2
3
4
5
Targeting vector
Mutant allele 1 (targeted allele)
R2
Mutant allele 2 (after Cre recombination)
5'
3'
Legends
UTR
Exon
loxP site
cKO region
Homology arm

sFig. 6 Impact of TREM2 deficiency on gene expression profile in the prefrontal cortex of T1D mice

a) Heatmap of top 50 up-regulated DEGs and top 50 down-regulated DEGs. **b)** PPI network of DEGs. **c)** Top 30 GO terms of DEGs. **d)** Top 30 KEGG terms of DEGs. **e)** Top 11 GSEA terms of hallmark gene sets. **f)** Gene expression heatmap of the PI3K/AKT/mTOR signaling pathway. **g)** Schematic of the gene targeting strategy for generating TREM2 cKO mice. T1D: Wild-type diabetic group. T1D+TREM2 cKO: TREM2 knockout diabetic group, PPI: protein-protein interaction, DEGs: Differentially expressed genes, GO: Gene ontology, KEGG: Kyoto encyclopedia of genes and genomes, GSEA: Gene set enrichment analysis.
